# Integrative omics analyses broaden treatment targets in human cancer

**DOI:** 10.1186/s13073-018-0564-z

**Published:** 2018-07-27

**Authors:** Sohini Sengupta, Sam Q. Sun, Kuan-lin Huang, Clara Oh, Matthew H. Bailey, Rajees Varghese, Matthew A. Wyczalkowski, Jie Ning, Piyush Tripathi, Joshua F. McMichael, Kimberly J. Johnson, Cyriac Kandoth, John Welch, Cynthia Ma, Michael C. Wendl, Samuel H. Payne, David Fenyö, Reid R. Townsend, John F. Dipersio, Feng Chen, Li Ding

**Affiliations:** 1 Division of Oncology, Department of Medicine, Washington University, St. Louis, MO 63108 USA; 2 McDonnell Genome Institute, Washington University, St. Louis, MO 63108 USA; 3 Division of Nephrology, Department of Medicine, Washington University, St. Louis, MO 63108 USA; 4 Brown School, Washington University, St. Louis, MO 63105 USA; 5 Marie-Josée and Henry R. Kravis Center for Molecular Oncology, Memorial Sloan Kettering Cancer Center, New York, NY USA; 6 Department of Mathematics, Washington University, St. Louis, MO 63108 USA; 7 Department of Genetics, Washington University, St. Louis, MO 63108 USA; 8 Biological Sciences Division, Pacific Northwest National Laboratory, Richland, WA 99352 USA; 9 Department of Biochemistry and Molecular Pharmacology, New York University Langone School of Medicine, New York, NY 10016 USA; 10 Institute for Systems Genetics, New York University Langone School of Medicine, New York, NY 10016 USA; 11 Siteman Cancer Center, Washington University, St. Louis, MO 63108 USA

**Keywords:** Cancer genomics, Multi-omics, Proteogenomics, Precision medicine, Cancer and druggability

## Abstract

**Background:**

Although large-scale, next-generation sequencing (NGS) studies of cancers hold promise for enabling precision oncology, challenges remain in integrating NGS with clinically validated biomarkers.

**Methods:**

To overcome such challenges, we utilized the Database of Evidence for Precision Oncology (DEPO) to link druggability to genomic, transcriptomic, and proteomic biomarkers. Using a pan-cancer cohort of 6570 tumors, we identified tumors with potentially druggable biomarkers consisting of drug-associated mutations, mRNA expression outliers, and protein/phosphoprotein expression outliers identified by DEPO.

**Results:**

Within the pan-cancer cohort of 6570 tumors, we found that 3% are druggable based on FDA-approved drug-mutation interactions in specific cancer types. However, mRNA/phosphoprotein/protein expression outliers and drug repurposing across cancer types suggest potential druggability in up to 16% of tumors. The percentage of potential drug-associated tumors can increase to 48% if we consider preclinical evidence. Further, our analyses showed co-occurring potentially druggable multi-omics alterations in 32% of tumors, indicating a role for individualized combinational therapy, with evidence supporting mTOR/PI3K/ESR1 co-inhibition and BRAF/AKT co-inhibition in 1.6 and 0.8% of tumors, respectively. We experimentally validated a subset of putative druggable mutations in BRAF identified by a protein structure-based computational tool. Finally, analysis of a large-scale drug screening dataset lent further evidence supporting repurposing of drugs across cancer types and the use of expression outliers for inferring druggability.

**Conclusions:**

Our results suggest that an integrated analysis platform can nominate multi-omics alterations as biomarkers of druggability and aid ongoing efforts to bring precision oncology to patients.

**Electronic supplementary material:**

The online version of this article (10.1186/s13073-018-0564-z) contains supplementary material, which is available to authorized users.

## Background

With the development of novel therapeutics and next-generation sequencing (NGS), medicine is entering an era in which cancer treatment can be tailored to the tumor molecular profile of the individual patient. While an increasing number of FDA-approved cancer drugs are paired with a companion diagnostic for mutational [1–3] or protein expression abnormalities [4], a given drug is often only considered for the cancer type (breast carcinoma, etc.) for which it was approved. Pan-cancer analyses have identified significantly mutated genes shared across cancer type subsets [5–7], suggesting the potential for treating patients based on the genetic profile of their tumor, regardless of cancer type. Efforts are underway to implement NGS in the clinical setting [8–11], and several studies have examined practical aspects of NGS implementation, such as use of FFPE tumor samples [12–14], concordance between NGS and other diagnostic platforms [15, 16], and quality assurance of variant calls [12–16] (Additional file 1). However, using tumor molecular profiles from NGS and other platforms to infer druggability is an ongoing challenge [12, 17, 18]. In particular, no systematic pan-cancer analysis has yet been conducted to explore the potential impact of comprehensive multi-omics for informing cancer therapy.

The Cancer Genome Atlas (TCGA), the Clinical Proteomic Tumor Analysis Consortium (CPTAC) [19], and other large-scale sequencing data sets represent an opportunity to identify “druggable” variants, i.e., variants that render a cancer type susceptible to a drug. A recent study quantified the percentages and types of cancers that may benefit from therapies traditionally used for other indications [17]. Although the general approach is promising and has important implications for clinical practice [20, 21], these efforts primarily use gene/drug interactions rather than mutation/drug interactions to infer druggability [12, 15, 17, 22]. None leverage transcriptomic and proteomic data in tandem with genomic profiles generated through TCGA. Moreover, none leverage the compendium of known mutation/drug interactions to either discover or validate putative mutation/drug interactions.

Here, we present an analysis of the full spectrum of putatively druggable alterations in 6570 TCGA tumors based on integrative omics approaches. We utilized known variant/drug interactions from several data sources with each variant associated with sensitivity or resistance to a drug in preclinical or clinical studies [20, 23–25] (Sun et al. [26], in revision, http://dinglab.wustl.edu/depo). We identified tumors with drug-associated mutations and found considerable opportunity for repurposing of drugs across cancer types. We used a structure-based computational tool [27–29] to identify putative druggable mutations based on proximity to known druggable mutations and experimentally validated a subset of putative druggable mutations in BRAF. We then analyzed druggability based on mRNA, protein, and phosphosite expression levels. To identify opportunities for combinational therapy, we examined co-occurring potentially druggable alterations across multiple data types in tumors. Finally, we used a large-scale drug screen to validate our approach for inferring druggability across human cancers. By applying and validating novel approaches for inferring druggability, this report shows that more tumors than previously thought may be susceptible to targeted therapy and provides a concrete path for using integrative omics analyses to guide precision cancer therapy.

## Methods

### Construction of Database of Evidence for Precision Oncology (DEPO)

DEPO (Sun et al. [26], in revision, http://dinglab.wustl.edu/depo) was created as an information knowledgebase to facilitate downstream analyses in our study. Druggable variants in DEPO were filtered such that each variant corresponded to one of several categories: single nucleotide polymorphisms or SNPs (missense, frameshift, and nonsense mutations), in-frame insertions and deletions (indels), copy number variations (CNVs), or expression changes. The vast majority of SNPs and in-frame indels in DEPO are unambiguous, e.g., BRAF V600E. To accommodate looser categories of genomic events, DEPO allows missense mutations for which the substituted base is not specified (e.g., BRAF V600). Similarly, for SNPs and in-frame indels in a given exon (e.g., EGFR exon 19 in-frame deletion), we used Ensembl to convert to a codon-mapped nomenclature (e.g., EGFR p.729-761 in-frame deletion) [30].

Each variant/drug entry in DEPO was paired with several annotations of potential interest to oncologists. These annotations were generally derived from DEPO’s source databases, then standardized to the nomenclature discussed here. *Tumor type* is included for each variant/drug entry because, with infrequent exception, a variant’s effect on a tumor’s response to a given drug has only been rigorously studied in one or only a few cancer type(s). For a variant/drug entry based on preclinical data, tumor type was either inferred from the xenograft or cell line, or left unspecified. As indicated previously, *variant* can be annotated in several ways for SNPs and indels. It could be either a specific mutation, a specific amino acid position with no specified amino acid change, or a range of amino acid/genomic positions. Copy number amplifications (CNA) and losses (CNL), high expression outliers in oncogenes, low expression outliers in tumor suppressors, and fusions that may lead to druggability are also included. *Effect* describes whether a variant correlates with increased sensitivity of a tumor to a drug or increased resistance of a tumor to a drug. *Level of evidence* describes the quality of data supporting a given variant/drug entry: preclinical, case reports, clinical trials, and FDA approved. Some of this information was mined from clinicaltrials.gov. *Drug class* was determined using a look-up table that was generated manually from DrugBank/NIHClasses (Additional file 2: Table S1). A given drug entry in DEPO could be associated with multiple drug families to allow for the possibility of combining therapies (e.g., dabrafenib [B-Raf inhibitor] and trametinib [MEK inhibitor] for BRAF V600E/K-mutant melanoma) and multi-targeted tyrosine kinase inhibitors (e.g., afatinib as a dual HER2 and EGFR inhibitor). Finally, each entry in DEPO is linked to a *PubMed ID*, which was used to manually curate any missing annotations.

If two variant/drug entries had identical annotations for tumor type and effect, the entry with the highest level of evidence was used in DEPO. Otherwise, if two variant/drug entries had non-identical annotations, both were included. DEPO is available as a web portal (http://dinglab.wustl.edu/depo), through which users can search for variant entries to obtain therapeutic information. The version used for this analysis was from February 2017.

### Pan-cancer cohort and cancer types

We conducted analyses of druggability across a pan-cancer cohort of 6570 TCGA tumor samples from 22 cancer types [31]. These cancer types consisted of adrenocortical carcinoma (ACC), bladder urothelial carcinoma (BLCA), breast adenocarcinoma (BRCA), cervical squamous cell carcinoma and endocervical adenocarcinoma (CESC), colon and rectal carcinoma (COADREAD), glioblastoma multiforme (GBM), head and neck squamous cell carcinoma (HNSC), kidney chromophobe (KICH), kidney renal clear cell carcinoma (KIRC), kidney renal papillary cell carcinoma (KIRP), acute myeloid leukemia (AML/LAML), low-grade glioma (LGG), liver hepatocellular carcinoma (LIHC), lung adenocarcinoma (LUAD), lung squamous cell carcinoma (LUSC), ovarian serous carcinoma (OV), prostate adenocarcinoma (PRAD), skin cutaneous melanoma (SKCM), stomach adenocarcinoma (STAD), thyroid carcinoma (THCA), uterine corpus endometrial carcinoma (UCEC), and uterine carcinosarcoma (UCS).

### Collection of mutations in pan-cancer cohort

Variant calls were obtained from the TCGA Genome Data Analysis Centers (GDAC), Data Coordinating Center (DCC), and previously published TCGA marker papers until the end of 2014 (https://cancergenome.nih.gov/publications). Variant calls were excluded if metastases or recurrent samples were present for samples that already had a primary tumor in the mutation annotation file (MAF). When necessary, we used UCSC’s liftOver with an Ensemble chain file to convert variants from NCBI36 to GRCh37. Annotation was done by VEP v77 on Gencode Basic v19 transcripts, using vcf2maf (https://github.com/mskcc/vcf2maf) to a single canonical isoform per gene. We followed strict quality control processes and excluded variants without both nucleotide changes and genomic positions and variants whose MAF genotypes did not match VCF genotypes after accounting for matched strand. We filtered large indels (> 100 bp) and complex indels, which are not supported by the MAF specification. To remove duplicate samples, we excluded samples with > 60% variant concordance with another sample, unless both samples had five or fewer total variants. Furthermore, we filtered common variants, defined as minor allele frequency > 0.05% in the Exome Variant Server or 1000G [32, 33] cohort that were not pathogenic or deleterious/damaging according to Clinvar [34] and SIFT/Polyphen [35, 36].

### Drug-associated mutations in pan-cancer cohort

We identified tumors in our pan-cancer cohort that harbored one or more drug-associated SNP or indel. Iterating through a mutation annotation format (MAF) file containing all variants in our pan-cancer cohort, we performed two actions for each entry in the MAF. First, we queried a hash table containing all druggable, unambiguous mutations in DEPO (e.g., BRAF V600E) and a separate hash table containing all druggable, ambiguous, single-residue mutations in DEPO (e.g., BRAF V600). Second, we queried several classes of mutations that occur in a specific exon or segment of a gene (EGFR exon 19 in-frame deletion). All mutation entries in the MAF **(**Synapse ID, syn12618789**)** that map onto an entry in DEPO are stored, along with the corresponding TCGA tumor ID and tumor type (Additional file 2: Table S2).

In some cases, DEPO contains multiple entries per gene/mutation pair to reflect possible druggability of a gene/mutation pair in more than one tumor type, or that it may confer an effect (e.g., sensitivity or resistance) that depends on tumor type or other therapeutic context. Multiple DEPO entries per variant were used to generate visualizations of druggability. For example, when visualizing “drug repurposing” across tumor types, a given mutation could be associated with > 1 “cancer-type-specific” tumor type, if a given gene/mutation pair had druggability information in DEPO in multiple tumor types at the same level of evidence. For each unique gene/mutation pair, the cancer types that had the highest levels of evidence for a drug were considered cancer type specific. All other cancer types are considered non-specific for a gene/mutation pair. For example, DEPO indicates that BRAF V600E-mutated THCA is sensitive to BRAF inhibitor; however, because a higher level of evidence exists for BRAF V600E druggability in SKCM, THCA is “off-label” or “cancer type non-specific.” When considering potential druggable events in the cancer-type-non-specific setting, the drug with the highest level of evidence found across all tumor types was used for a specific variant (Additional file 2: Table S3). For downstream analyses (i.e., protein structure-based clustering, co-occurring mutation analysis, and integration analysis), variant/drug interactions were considered in this cancer-type-non-specific setting. If any sensitive interaction for a variant was found regardless of the tumor type and level, it was considered a “druggable” event for these analyses. Additionally, if there was evidence for both resistant and sensitive drug interactions for a specific variant, the sensitive interaction was utilized.

### Proximity-based clustering of drug-associated mutations with pan-cancer cohort

HotSpot3D [27] was used to spatially cluster “known” drug-associated mutations in DEPO with putative druggable mutations in our pan-cancer cohort. In brief, pairwise distances between all amino acids are calculated to give a background distribution. We assigned a *P* value to the pairwise distance and defined it as the proportion of all pairwise amino acid 3D distances that are less than or equal to the distance between the pair of amino acids in question. After this, we only performed clustering on significant pairs having *p* < 0.05 and distance less than 5 Å.

Single-link agglomerative clustering forms initial clusters from the significant proximal pairs by iteratively adding new mutations to a cluster if they are significantly paired with a mutation already in the cluster. To prevent a cluster with unbounded size, we applied a limit to the physical extent of the clusters. If the initial cluster is modeled as an undirected graph *G* = (*V*, *E*), where *V* is the set of all mutations in the initial cluster and *E* is the set of 3D distances of all proximal pairs in *V*, we can calculate the shortest path from each vertex to all other vertices. We identify a centroid of the cluster to be the mutation that is found more frequently in patient samples as well as the one found in close proximity to highly recurrent mutations. The clusters are then focused according to a specified graph radius limit from the centroid.

The original clustering approach for HotSpot3D was improved upon in this analysis by using recursive clustering. Briefly, setting a maximum radius limit could lead to potentially functional regions being ignored. To bypass this problem, instead of discarding mutations outside of the radius limit, we performed clustering on the remaining mutations in the initial cluster. We continued to do this until no more clusters could be found. For this analysis, a radius limit of 5 Å was used in order to limit clusters to a relatively conservative size. We did not use a linear distance limit in order to detect all mutations that cluster closely to drug-associated mutations, regardless of position on amino acid sequence.

### Druggable expression outliers in pan-cancer cohort

RNA expression data (TCGA level 3, normalized) were downloaded from firehose (October 17, 2014). We log_2_-transformed the RNA-seq by expectation-maximization (RSEM) values of RNA expression data for outlier analysis. RPPA data (level 4, normalized) were downloaded from The Cancer Protein Atlas (TCPA) and were normalized across batches using replicates-based normalization (RBN) as previously described [37].

To discover expression outliers, we utilized a strategy incorporating multiple steps. First, we limited our search to genes in DEPO whose overexpression or copy number amplification is associated with drug sensitivity; these tended to be proto-oncogenes. We then narrowed down the list to genes that are observed in at least 10 tumor samples in the dataset under investigation. Additionally, we did not include AML in our expression analysis. Outlier expressions were defined as values that are greater than 1.5 interquartile ranges (IQRs) above the third quartile (Q3), or below the first quartile (Q1) across the pan-cancer cohort. To rank order outlier expression for each gene, we calculated an outlier score defined as:

$$ {\displaystyle \begin{array}{c}\mathrm{Outlier}\ \mathrm{score}=\left(x-\mathrm{Q}3\right)/\mathrm{IQR}\\ {}\mathrm{or}\\ {}\mathrm{Outlier}\ \mathrm{score}=\left(\mathrm{Q}1-x\right)/\mathrm{IQR}\ \end{array}} $$

By definition, genes with outlier score greater than 1.5 are considered as expression outliers. Outlier score for each gene were ranked within each tumor sample to select the most promising “druggable” targets.

Only RNA-seq and RPPA data was utilized for all subsequent analysis and calculating potential druggable targets for transcriptomic and proteomic expression outliers.

### Fusion analysis

Fusions were obtained from a prior publication [31] that identified fusion transcripts in 4366 tumors. We restricted our analysis to the intersection between the 4366 tumors in Yoshihara et al. and the 6570 tumors assessed in the present study. Only fusion transcripts corresponding to a druggable fusion gene in DEPO were considered in constructing Additional file 3: Figure S1. To correlate fusion transcripts and expression, we identified RNA and phosphoprotein expression levels (outlier scores) for druggable fusion genes (Additional file 3: Figure S1).

### Proteomic analysis with CPTAC mass spectrometry data

The 251 Clinical Proteome Tumor Analysis Consortium (CPTAC) tumors used in our analysis included 77 breast cancer tumors [38], 90 colorectal cancer tumors [39], and 84 ovarian cancer tumors (from PNNL only) [40]. Proteomic data were processed using the Common Data Analysis Pipeline [41]. Analysis was conducted with this data to reveal potential druggable proteomic outliers in the three cancer types (Additional file 1, Additional file 3: Figure S2); however, these numbers were not included in our subsequent analyses or our summative assessment of pan-cancer druggability.

### Cell line-based validation

Cell line data was downloaded from the Genomics of Drug Sensitivity in Cancer (GDSC) database (http://www.cancerrxgene.org/downloads). Specifically, the data of interest were the screened compounds (Additional file 2: Table S4), log(IC50) and AUC values, the expression array data for cell lines, and the WES data for cell lines. The first step was to convert DEPO drug names into the drug IDs provided in the screened compounds. We were inclusive in terms of matching drugs from the cell line data to DEPO, so that we would have enough statistical power and data points to study trends. The drug ID for the screened compound was included for a DEPO drug if one of the following were satisfied: (1) drug name in DEPO matched exactly the drug name or synonym in screened compounds from the cell line data and (2) the gene target of the drug class/drug in DEPO matches the gene target of the drug in screened compounds. Additionally, the list was refined through manual manipulation.

For mutation analysis, cell lines that contained mutations in DEPO were analyzed for their LN(IC50) values. These mutations were separated into cancer-type-specific and non-specific if the cancer type of the cell line did not have the highest level of evidence in DEPO for a specific mutation (Additional file 2: Table S5). Similar to our mutation analysis of TCGA data, the drug with the highest level of evidence for a particular mutation was used (Additional file 2: Table S3). The distribution of LN(IC50) values of cell lines with DEPO mutations (both sensitive and resistant) for both the cancer-type-specific and non-specific settings were compared to a background distribution using the Mann-Whitney *U* test. The background distribution consists of all LN(IC50) values from every drug-cell line combination whether they have a DEPO mutation or not. In addition to comparing overall distributions, we also compared distributions of LN(IC50) for cell lines with a specific sensitive mutation to the distribution of LN(IC50) values across all cell lines for the particular drug in question (Additional file 2: Table S6). This was done in both the cancer-type-specific and non-specific settings. We required that there be at least five cell lines that contain the specific sensitive mutation A tested against drug B in order to deem significance of the drug-mutation combination.

For expression analysis, Affymetrix Human Genome U219 array data from ArrayExpress (E-MTAB-3610) were used. The expression data were in the form of an Affymetrix *CEL* Data File, which required conversion to a gene expression matrix in order to run through the expression outlier analysis pipeline. This was done using Bioconductor in R and the “affy” Library. The file was then annotated with genes using an annotation package (hgu219.db) through Bioconductor. The resulting matrix was run through the outlier expression pipeline detailed above. Genes that were known to confer drug sensitivity through expression based on DEPO were analyzed. Each gene could have multiple probes, and all probes were included in downstream analysis. To test whether gene expression is correlated with drug sensitivity, we conducted linear regressions on all probe-drug combinations in the form of *y*_*i*_ = *Bx*_*i*_ + *a*, where *x*_*i*_ is the gene expression outlier score for a specific gene probe in cell line *i* and *y*_*i*_ is the LN(IC50) value for a drug associated with the gene in cell line *i.* There were 496 probe-drug combinations with sufficient sample size, at least five samples, to conduct regression analysis (Additional file 2: Table S7). Probe-drug combinations that had *P* < 0.05 and *B* < 0 were considered to have a significant correlation between gene expression and drug sensitivity.

In reporting potential druggability across the TCGA cohort, we considered all tumors with mutational evidence; however, we only considered tumors with mRNA and protein/phosphoprotein outliers for genes that could be validated against GDSC data regardless of level of approval. A gene was considered to be “validated” if at least one of its probes had a significant *P* value for the regression between gene outlier score and LN(IC50) and these two variables were negatively correlated.

### Experimental validation

HEK293T cells were authenticated by DNA finger printing targeting short tandem repeat (STR) profiles through Genetica Cell Line Testing. They are negative for mycoplasma as determined by the absence of extranuclear signals in DAPI staining. Cells were cultured in DMEM (Corning) supplemented with 5% fetal bovine serum (FBS) (Thermo Fisher). Constructions expressing BRAF variants were generated from a plasmid expressing a wild-type BRAF (Addgene, #40775) with an N-terminal Flag tag using Q5 site-directed mutagenesis (New England BioLabs). All constructs were confirmed by sequencing. Cells were transiently transfected with wild-type or mutant BRAF constructs using Lipofectamine 2000 reagent (Life Technologies) in six-well plates. Twenty-four hours after transfection, cells were switched to medium containing 0.5% FBS for 24 h before the initiation of 6 h of treatment with Dabrafenib (0–1 uM). Cells were lysed in buffer containing 20 mM Tris-HCl (pH 7.5), 150 mM NaCl, 1 mM Na2EDTA, 1 mM EGTA, 1% NP-40, 1% sodium deoxycholate, 2.5 mM sodium pyrophosphate, 1 mM β-glycerophosphate, 1 mM sodium orthovanadate, and 1 μg/ml leupeptin (Cell Signaling Technology). Protease and phosphatase inhibitors (Roche) were added immediately before use. Samples (15 μg/lane) were boiled in standard commercial SDS-gel loading buffer and run on SDS 10% polyacrylamide gels. Immunoblotting was performed on Immobilon-P PVDF membrane (Millipore). The following antibodies were used for immunoblotting: rabbit polyclonal anti-phosphor-MEK1/2 (Ser217/221) antibodies (Cell Signaling #9121, at 1:1000 dilution), mouse monoclonal anti-MEK1/2 antibodies (Santa Cruz, sc-81504, at 1:500 dilution), mouse monoclonal anti-Flag antibodies (Sigma-Aldrich F1804, 1:1000), and rabbit polyclonal anti-GAPDH antibodies (Cell Signaling, #5174, at 1:1000 dilution). Appropriate secondary antibodies with infrared dyes (LI-COR) were used. Protein bands were visualized using the Odyssey Infrared Imaging System (LI-COR) and further quantified by ImageJ.

### Integrative omics analysis of druggability

To analyze and visualize druggability based on multi-omics information, we first identified tumors whose druggability is implicated by two or more variant types (genomic, transcriptomic, proteomic). Drug-associated genomic variants include both known mutations in DEPO and putative mutations identified using protein structure-based clustering. Transcriptomic and proteomic variants include mRNAs and phosphoproteins/proteins with expression outliers based on RNA-seq and RPPA data, respectively. For each tumor, we mapped its “druggable” variants against one or more drugs, which were then mapped to one or more drug classes (Additional file 2: Table S8). For each variant, we used the drug that had the highest level of evidence in DEPO regardless of cancer type **(**Additional file 2: Table S3). For the purposes of visualization, we only considered ten FDA-approved drug classes **(**Additional file 2: Table S9) mapping to the largest number of variants across our pan-cancer cohort (Additional file 2: Table S10).

### Druggability and demographics

We assessed differences in druggability as a function of demographics (sex, race) (Additional file 1, Additional file 2: Table S11, Additional file 3: Figure S4). We limited our analyses to cancer types for which at least 20 tumors are represented for each demographic category (e.g., ≥ 20 Caucasians with BRCA, ≥ 20 Asians with BRCA). For the sex analysis, this excluded certain cancer types (BRCA, CESC, PRAD, OV, UCEC, and UCS). Next, we determined the most commonly druggable genes at the mutational, RNA, and phosphoprotein levels; to merit inclusion, a druggable gene must be observed in ≥ 40 tumors and ≥ 150 tumors for the race and sex analyses, respectively. A matrix was then generated of cancer types and druggable genes, with each matrix value corresponding to the log-odds ratio between druggability and traits:

$$ {\log}_2\left(\frac{\mathrm{druggable}\ \mathrm{trait}\ \mathrm{A}\ \mathrm{patients}/\mathrm{trait}\ \mathrm{A}\ \mathrm{patients}}{\mathrm{druggable}\ \mathrm{trait}\ \mathrm{B}\ \mathrm{patients}/\mathrm{trait}\ \mathrm{B}\ \mathrm{patients}}\right) $$

for a specific cancer type (e.g., BRCA) and a specific druggable gene (e.g., elevated ERBB2 phosphoprotein expression). If fewer than 10 tumors contain a specific druggable gene in a specific cancer type, no matrix value was calculated. For the purposes of graphical visualization, matrix values of +∞ and –∞ are set to + 3 and − 3, respectively.

To determine whether a specific druggable gene is statistically more prevalent in a given demographic group, Fisher exact tests were performed. FDR correction to *p* values was applied with a cutoff of 0.05.

## Results

### Database of Evidence for Precision Oncology

**Fig. 1:**
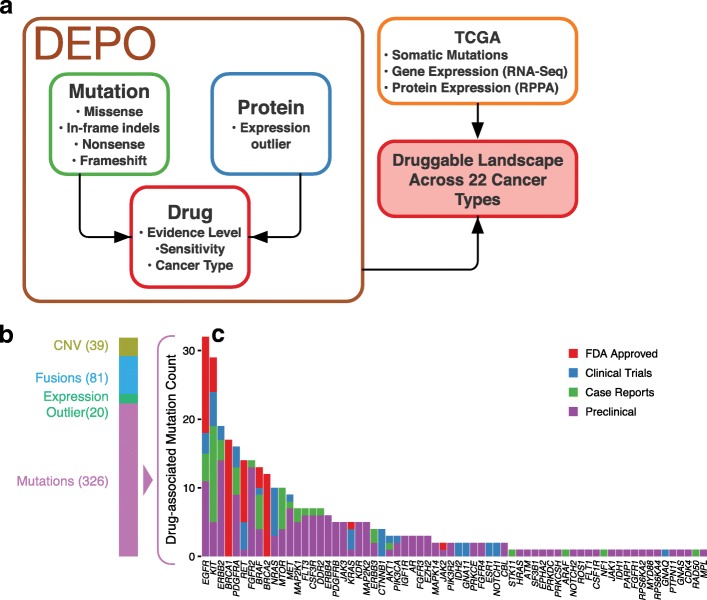
DEPO database. **a** The methodology supporting curation of the drug-variant depository, which we refer to as DEPO, or *D*atabase of *E*vidence for *P*recision *O*ncology, and its use in determining the “druggable” landscape of TCGA tumors. **b** The composition of sensitive variants in DEPO by variant type. For each variant type, only unique variants were counted even if a given variant is associated with multiple levels of evidence, multiple drugs, and/or multiple cancer types. “CNV” (copy number variation) corresponds to “CNA” (copy number amplification) and “CNL” (copy number loss) entries in DEPO; this includes genes for which CNA or CNL is associated with drug response, respectively. “Expression” refers to genes whose elevated and reduced expression is associated with drug response. “Mutations” refers to missense, nonsense, in-frame indels, and frameshift mutations. **c** Number of uniquely drug-associated mutations in DEPO by gene, sorted by evidence level: FDA approved, clinical trials, case reports, and preclinical

We utilized a repository of known variant/drug interactions, which we refer to as “Database of Evidence for Precision Oncology” or DEPO (Sun et al.[26], in revision), containing data from publically available datasets and papers [20, 23–25] (Fig. 1a).

In aggregate, 609 unique variants with known drug interactions currently reside in DEPO, and account for a total of ~ 800 unique variant/drug interactions (Fig. 1b). Approximately 70% of known variant/drug interactions result in increased sensitivity to therapy. Further, a substantial number (~ 25%) of sensitive variant/drug interactions are approved by the FDA for a particular cancer type or are based on late-stage clinical studies. Several genes account for a large proportion of variant/drug interactions (e.g., *EGFR*, *KIT*, *ERBB2*, *BRCA1*, *PDGFRA*), reflecting interest in therapeutically exploiting a relatively limited number of cancer driver genes [5] (Fig. 1c). Altogether, 168 genes are represented in the current version of DEPO.

### Drug-associated mutations in pan-cancer cohort

We leveraged the genomic sequence data of 6570 tumor samples from TCGA representing 22 adult cancer types (Synapse ID, syn12618789). Mutations associated with drug sensitivity in DEPO were matched against the TCGA cohort. Our analysis reveals 2364 mutations across 2114 tumors that are associated with sensitivity to one or more drugs (mean = 1.12/tumor) (Additional file 2: Table S2). Three hundred sixty-two distinct mutations are represented across 40 genes. The low fraction of drug-associated mutations likely reflects the large number of passengers in cancer [42, 43]. Thirty-two percent of tumors had at least one drug-associated mutation, a percentage that is consistent with the 28% of screened patients that could be matched with a targeted therapy or trial [44].

**Fig. 2:**
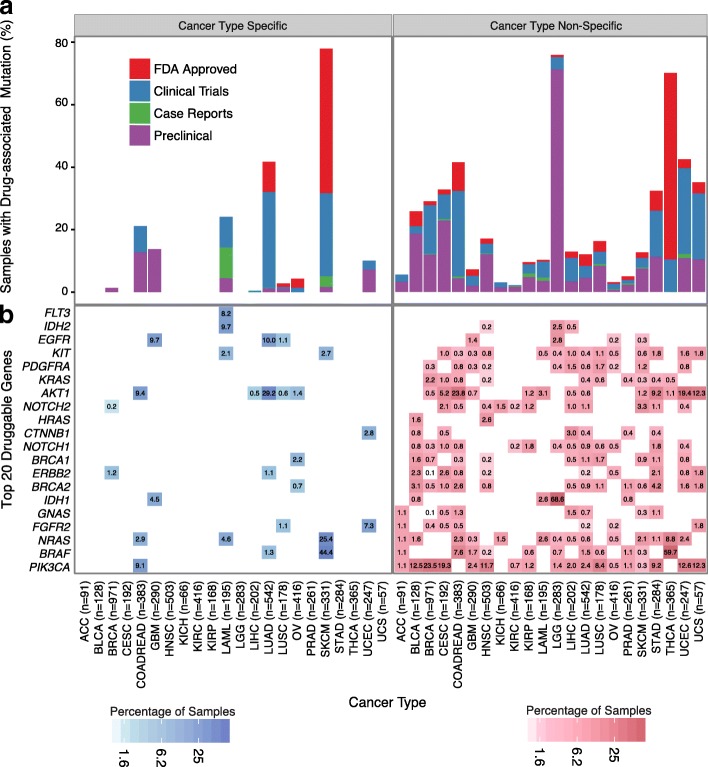
Drug-associated mutations across cancer types. Both **a** and **b** can be broken down into cancer-type-specific and cancer-type-non-specific settings. **a** Fraction of tumors (*y*-axis) for a given cancer type (*x*-axis) that have at least one drug-associated mutation. Both bar graphs are sorted by evidence level. For the cancer-type-specific graph, only the cancer types with the highest level of evidence per mutation are shown. For the cancer-type-non-specific graph, the highest level of evidence available for each mutation independent of cancer type is used, which is derived from the cancer-type-specific setting. **b** Fraction of tumors (intensity of shading) for a given cancer type containing a drug-associated mutation from a specific gene (*y*-axis). Only the top 20 genes with drug-associated mutations present in the largest number of tumor samples across the TCGA cohort are displayed

Initially, we analyzed the percentage of potentially druggable tumors in a cancer-type-specific setting (Fig. 2), that is, tumors with mutations associated with a known drug response in the cancer type with the highest level of evidence. Only 3.3% of the samples contain a druggable mutation known to be FDA approved; however, if we consider less mature evidence: clinical trials, preclinical, and case reports, we could potentially increase the percentage of tumors with drug-associated mutations to 8.2, 8.5, and 10.5%, respectively. Here, skin cutaneous melanoma (SKCM) is the cancer type with the largest fraction of drug-associated mutations (78%). SKCM with a BRAF V600E/K mutation (40% of patients) can be treated with BRAF and MEK inhibitors based on FDA approval. The NRAS Q61 mutations found in 12% of SKCM patients are more challenging to treat, as is any RAS-mutant cancer due to activation of multiple signaling pathways. Early generation MEK-exclusive inhibition proved to be ineffective, with multiple failed clinical trials prompting exploration of newer generation MEK inhibitors and MEK inhibitor combinations with downstream targets of NRAS [45]. In colon and rectal carcinoma (COADREAD), glioblastoma multiforme (GBM), and lung adenocarcinoma (LUAD), 21, 14, and 40% of their respective tumors contain a drug-associated mutation in a cancer-type-specific setting. In COADREAD, drug-associated variants PIK3CA E542K, E545K, and H1047R are present in 2.1, 5.2, and 1.8% of tumors, respectively, and are associated with sensitivity to PI3K/AKT/mTOR pathway inhibitors in early-stage trials [46] and aspirin in observational studies [47, 48]. PIK3CA*-*mutant cancers are also an ongoing challenge to treat clinically; co-occurring drugs targeting the PI3K pathway have been more effective than single-agent PI3K inhibition in treating PIK3CA-mutant cancers, but efficacy varies with mutation profile [46]. In GBM, the EGFR extracellular mutations (A289V, G598V, and R108K) and *IDH1* mutation R132H are present in 10 and 4.5% of tumors, respectively, and are associated with drug response based on preclinical data [49]. In non-small cell lung cancer, EGFR inhibitors (e.g., erlotinib) are FDA approved for tumors with activating EGFR mutations, which are present at 10 and 1% in our LUAD and lung squamous cell carcinoma (LUSC) cohorts, respectively.

**Fig. 3:**
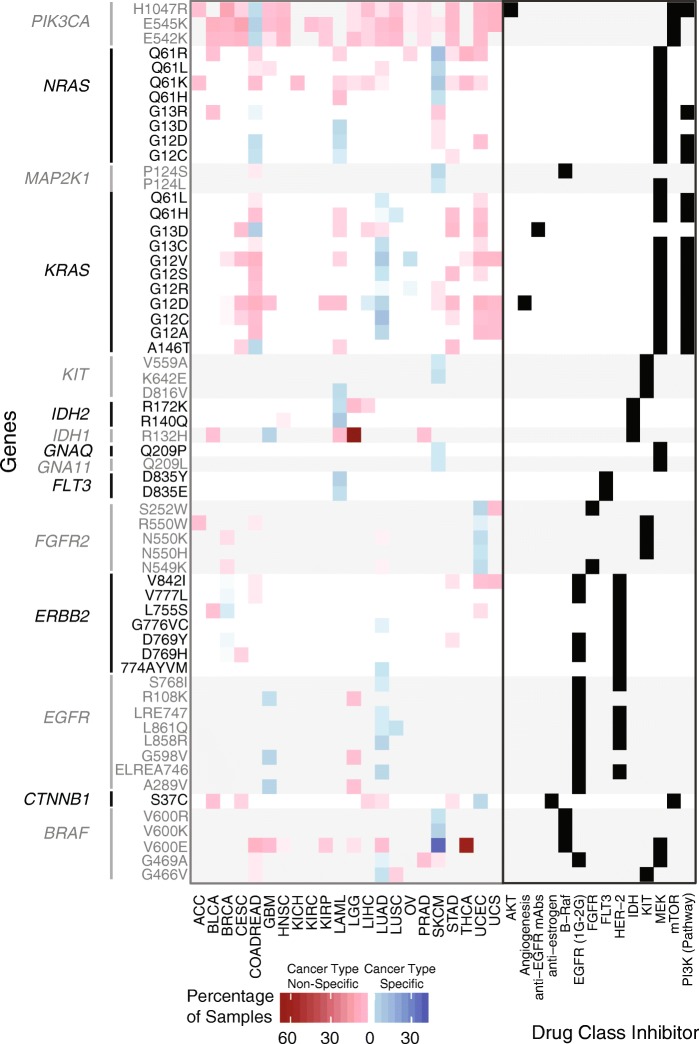
Repurposing of drugs using common mutations associated with drug sensitivity. Cancer-type-specific mutations (blue) and cancer-type-non-specific mutations (red) are distinguished. Intensity of shading corresponds to the fraction of tumors for a given cancer type (*x*-axis) that contain a specific drug-associated mutation (*y*-axis). Drug classes associated with each cancer-type-specific mutation from DEPO are shown in the right panel. Only drug-associated mutations present in the largest number of tumor samples across the TCGA cohort are displayed

Despite the promise of targeted therapy, only 10.5% of this pan-cancer cohort contains potential drug-associated mutations in a cancer-type-specific setting. With drug repurposing across cancer types, in which a drug used primarily in cancer type A with mutation X is repurposed for cancer type B with mutation X, we find that an additional 5.4% of patients may be treated with a FDA-approved drug-variant interaction (Figs. 2 and 3, Additional file 2: Table S12). This number can be increased to 22.8% if we consider repurposing of lower tier drug-variant pairs to other cancer types; however, these interactions will require clinical validation to be considered truly druggable. In this cancer-type-non-specific setting, cancer types in which at least 40% of tumors have drug-associated mutations include low-grade glioma (LGG, 76%), thyroid carcinoma (THCA, 70%), and colorectal adenocarcinoma (COADREAD, 42%). A small number of drug-associated mutations occur at high frequency in these cancer types. For example, in THCA, the BRAF V600E variant is found in 60% of tumors. Clinical trials have investigated the use of BRAF inhibitors combined with MEK inhibitors in THCA. However, *BRAF* V600E also occurs at a lower frequency in HNSC, KIRP, LGG, and GBM, indicating significant repurposing potential for BRAF inhibitors [50, 51] (Fig. 3).

COADREAD may also have potential for therapeutic intervention via repurposing (Fig. 2a). However, COADREAD has been difficult to treat due to a large presence of KRAS and BRAF mutations; EGFR inhibition as monotherapy is used for COADREAD, but only in tumors with wild-type KRAS [52, 53]. Repurposing drugs that inhibit downstream effectors of KRAS (e.g., MEK) is an alternative therapeutic strategy for KRAS-mutant COADREAD (23.8% of patients). The efficacy of MEK inhibition in combination with sorafenib has been tested in clinical trials for KRAS- or NRAS-mutant liver hepatocellular carcinoma (LIHC) [54] and has shown positive results. Co-targeting of MEK and AKT signaling showed some durable response in a phase I study [55], and most recently, a small trial showed some success combining an investigational MEK inhibitor with a CDK4/6 inhibitor in non-small cell lung cancer (NSCLC) (trial NCT number NCT02022982). COADREAD or other cancer types having RAS mutations, such as cervical squamous cell carcinoma and endocervical adenocarcinoma (CESC), acute myeloid leukemia (AML), stomach adenocarcinoma (STAD), and uterine corpus endometrial carcinoma (UCEC), could benefit from further exploration of combinatorial therapies targeting downstream targets of KRAS (Fig. 2b). BRAF-mutant COADREAD (7.6% of patients) presents a similar problem in that BRAF inhibitor monotherapy is ineffective unlike in BRAF-mutant melanoma and that triple drug combination targeting the EGFR, MAPK, and PI3K pathway has shown more positive results. Numerous clinical trials are underway to find the best combination therapies with BRAF inhibitors, including new drugs that are Wnt pathway and cyclin-dependent kinase inhibitors [56]. Together, cancer-type-specific and non-specific mutational analyses identified potential therapeutic targets in 2114 tumors (32%), some of which will be considered druggable only with further clinical development and FDA approval.

### Protein structure-based clustering of drug-associated mutations

**Fig. 4:**
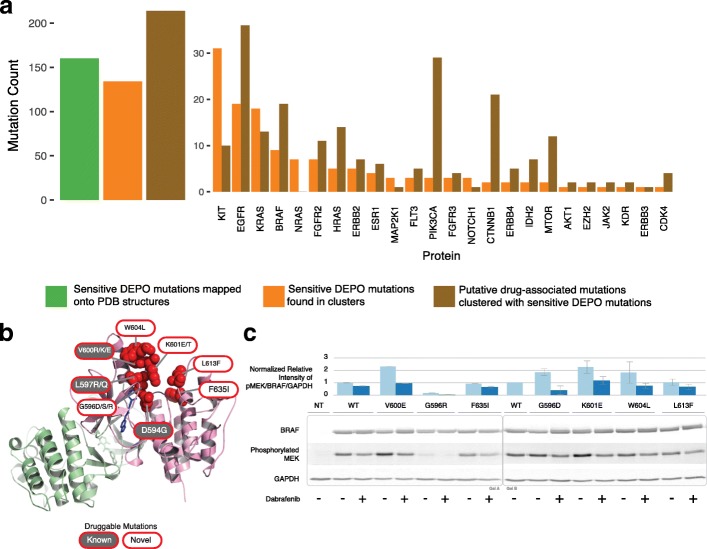
Protein structure-based analysis of drug-associated mutations. **a** The number of known drug-associated mutations that can be mapped onto PDB structures, the number of known drug-associated mutations that are found in HotSpot3D clusters, and the number of putative druggable mutations are shown, both in aggregate and for specific genes (*x*-axis). **b** Protein structure views of one HotSpot3D cluster in BRAF (PDB: 4MBJ). Known and putative druggable mutations are distinguished by different colors in mutation labels. A drug molecule in the binding pocket is indicated in blue. **c** Western blot for BRAF mutation cluster found in **b**. HEK293T cells were transiently transfected with wild-type (WT) or mutant BRAF constructs and were cultured in 0.5% calf serum for 24 h before treatment with Dabrafenib (0-1uM) for 6 h. BRAF activity was analyzed by quantifying phosphorylation changes in MEK1/2. To normalize for transfection and loading variations, pMEK levels were divided by BRAF levels and then by GAPDH levels to produce the normalized relative intensities of pMEK/BRAF/GAPDH. This was then normalized to the WT sample without drug treatment that was set as 1. The error bars represent biological replicates

We applied a structure-based clustering tool, HotSpot3D [27], to the pan-cancer dataset to reveal putative functional mutations (Additional file 2: Table S13). HotSpot3D’s utility in predicting functional mutations is supported by experimental evidence using cell lines expressing one of several EGFR-mutant proteins [36]. HotSpot3D identifies mutations that, by clustering in protein space with mutations from DEPO associated with drug sensitivity or resistance, may themselves affect drug binding affinity and response. Out of 160 “sensitive” mutations from DEPO that mapped onto protein structures, we identified 134 “sensitive” mutations in HotSpot3D clusters, which in turn were clustered with 214 putative sensitive mutations that were not catalogued in DEPO. These mutations were found in 55 clusters from 24 genes (Fig. 4a). Among all genes in our analysis, EGFR contains the highest number of putative sensitive mutations, with 36 mutations that clustered with 19 mutations in DEPO from seven different clusters (Fig. 4a). This clustering analysis helps winnow down the mutation list to candidates likely to affect drug response and provides context for further experimental testing, but does not necessarily indicate the direction of drug response; in total, HotSpot3D analysis identified potential therapeutic targets in 458 tumors (7%).

We identified putative resistant mutations as those that clustered with “resistant” mutations from DEPO; further, to prevent contradictory annotation of putative mutations as both “sensitive” and “resistant,” we limited our analysis of clusters containing “resistant” mutations to those that did not overlap with clusters containing sensitive mutations. This procedure yielded four different clusters with a “resistant” mutation in AKT1, MAP2K1, and *RAC1*; these four clusters contained 14 putative resistant mutations clustering with four known resistant mutations **(**Additional file 2: Table S13). RAC1 yielded the largest cluster, with RAC1 P29S mediating resistance to BRAF inhibitors in BRAF-mutant SKCM [57]. Other mutations in this cluster that may affect binding affinity of BRAF inhibitors (or that may mediate resistance to BRAF inhibitors) are C18Y, E31D, A159V, P29L/T, and P34S.

To provide evidence in support of mutation clustering as a method for identifying putative druggable mutations, we first show that known drug-associated mutations in DEPO that affect binding affinity of drugs in the same drug class cluster spatially. Most clusters contain more than one known drug-associated mutation. For example, KIT has multiple clusters with known mutations; one of which has three known mutations (E490D, Y494C, S476G) in the same cluster, which are FDA approved as sensitive to combined therapy of imatinib, sunitinib, and regorafenib (KIT and angiogenesis inhibitor). In addition, this cluster contains two other unique mutations (D439H, I438L) not in DEPO that, based on our analysis using HotSpot3D, could also affect binding affinity and potentially tumor sensitivity to KIT combined with angiogenesis inhibitors (Additional file 2: Table S13). Second, we experimentally validated HotSpot3D as a tool for identifying functional mutations associated with drug response. To do this, we assessed the activity and drug sensitivity of a set of six BRAF mutations (F635I, G596D, K601E, W604L, L613F, G596R) in close spatial proximity to the well-studied V600E pathogenic mutation (Fig. 4b). A key function of BRAF is phosphorylating MEK1/2. Therefore, we transfected BRAF mutations, along with wild-type BRAF and BRAF V600E, into HEK293T cells in the presence or absence of BRAF inhibitor dabrafenib, and used phosphorylation changes in MEK1/2 as an indicator of BRAF activity. The undetectable level of endogenous BRAF in HEK293T cells eliminates potential ambiguity in interpreting the effects of transfected BRAF mutations. As expected, BRAF V600E caused drastically increased phosphorylation in MEK1/2 that is reduced by dabrafenib (Fig. 4c). Three (G596D, K601E, and W604L) out of six other transfected BRAF mutations also showed higher levels of MEK1/2 phosphorylation and sensitivity to dabrafenib than wild-type BRAF, suggesting that a high percentage of mutations identified by Hotspot3D in close spatial proximity to V600E are activated and similarly sensitive to dabrafenib. Notably, BRAF G596R-transfected cells appeared to have a much lower level of MEK1/2 phosphorylation when compared to those transfected with wild-type BRAF, supporting prior findings that G596R results in BRAF loss of function [58]. Our ongoing development of comprehensive computational tools combining spatial proximity with considerations of specific amino acid substitutions and other structural features will further improve the accuracy of identifying functional mutations. Overall, HotSpot3D, combined with experimental assays, can help identify functional mutations that are candidates for inclusion in DEPO and worth further clinical exploration.

### Druggable gene and protein expression outliers in pan-cancer cohort

In addition to driver mutations in oncogenes, elevated expression of genes or gene products can also be used to select tumors for targeted therapy [59–61]. For example, in the case of breast cancer, elevated mRNA expression and copy number amplification of ESR1 correlate with elevated protein expression of ER [62, 63], as well as with sensitivity to hormonal therapy with tamoxifen [62, 64]. In general, tumors with elevated protein expression may respond to drugs that activate antibody-dependent cell-mediated cytotoxicity [65], suppress signaling pathways essential for tumor survival [66], or deliver cytotoxic agents via tumor-specific antigens [67].

**Fig. 5:**
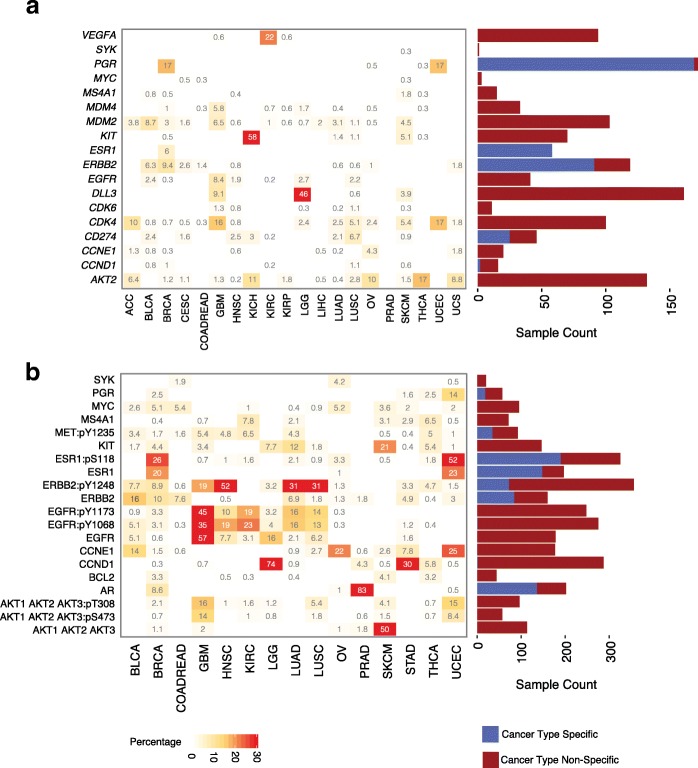
Druggable gene and protein expression outliers. Outlier expression analysis for mRNA (**a**) and protein and phosphoproteins (**b**) in TCGA tumors. Intensity of shading corresponds to percentage of tumor samples in a specific cancer type (*x*-axis) that has outlier expression in a specific gene (*y*-axis). The scale is limited to 30%; any percentage higher than this will be displayed as the same color. The bar graphs show how many tumors have outlier expression in each specific gene. Blue refers to potential druggable cancer-type-specific tumors and maroon refers to potential druggable “cancer-type-non-specific” tumors. In **b**, protein and phosphoproteins are represented, with phosphoproteins distinguished by a “:” followed by the phosphorylation site

Therefore, to further expand the set of tumors with potential drug-associated biomarkers, we sought transcriptomic and proteomic evidence of elevated gene/protein expression. For each gene in DEPO whose expression is associated with drug response, tumors with outliers were identified using the pan-cancer cohort as a reference. We defined outliers as expression values exceeding 1.5 interquartile ranges (IQR) above the third quartile of the cohort [68]. We applied this outlier detection strategy across mRNA, protein, and protein phosphorylation levels. RNA-seq and protein RPPA data are available for 5286 and 3877 tumors out of 6570 tumors in the TCGA cohort, respectively (Additional file 2: Table S14). DEPO has 50 genes whose expression is associated with drug response, 39 of which are associated with drug sensitivity. We identified elevated expression of druggable genes with drug sensitivity in 16 and 30% of the pan-cancer cohort of 6570 TCGA tumors at the mRNA and protein/phosphoprotein levels, respectively (Fig. 5). Interestingly, tumors with “druggable” gene fusions tend to express elevated levels of the corresponding druggable gene (Additional file 2: Table S15, Additional file 3: Figure S1) [69], suggesting that fusions may be one of several drivers of gene and protein expression.

To determine mRNA expression outliers in tumor samples, we used RNA-seq data from TCGA (Fig. 5a). Elevated DLL3 expression was identified in 161 tumors, including LGG, GBM, and SKCM tumors. DLL3 contributes to neuroendocrine tumorigenesis by inhibiting the Notch signaling pathway, whose role is to suppress tumor growth. A DLL3-targeted antibody-drug conjugate in phase II clinical trials effectively targets DLL3-expressing cells in high-grade pulmonary neuroendocrine tumors [70, 71]. This same therapy could potentially benefit GBM, LGG, and SKCM via repurposing due to shared levels of high DLL3 expression. Seventeen percent of BRCA and UCEC express PGR and 9.4% of BRCA express ERBB2 in our cohort, reflecting the FDA-approved use of anti-estrogen hormone therapy and HER-2 inhibitors, respectively, in these cancer types. ERBB2 is expressed in other cancer types, such as BLCA and CESC, which could benefit from repurposing and further exploration of HER2-inhibition; HER-2 inhibitors for COADREAD are currently being explored in late-stage clinical trials.

To examine tumors with potential drug-associated biomarkers based on protein expression and phosphosite levels, we used TCGA reverse phase protein array (RPPA) data (Fig. 5b). Compared to the pan-cancer cohort, 83% of prostate adenocarcinoma (PRAD) express elevated AR, reflecting their tissue of origin. Elevated AR is also present in 9% of breast adenocarcinoma (BRCA). These 9% of BRCA express higher levels of AR than 17% of PRAD, suggesting that androgen-deprivation therapy can potentially be repurposed for AR-positive BRCA [72] (Additional file 2: Table S16). Similarly, 26 and 52% of BRCA and UCEC, respectively, show elevated activity at ESR1’s p.S118 phosphosite. These only represent a fraction of druggable BRCA, as 77% of tumors in a large breast cancer registry are ER positive [73]. Elevated expression and activity of EGFR protein and its phosphosites across cancer types suggest that phosphoproteome analysis may inform treatment response. EGFR phosphosites p.Y1068 and p.Y1173 are active in GBM, head and neck squamous cell carcinoma (HNSC), KIRC, LUAD, and LUSC. Some evidence has shown that HNSC, LUAD, and LUSC are responsive to EGFR tyrosine kinase inhibitors (TKIs) [74, 75], perhaps because EGFR TKIs inhibit autophosphorylation rather than elevated protein expression [76]. In KIRC, EGFR inhibitors have negligible activity [77–79] despite active phosphosites in our analysis, possibly because EGFR is one of many growth factors expressed in KIRC or because EGFR inhibition is ineffective in the absence of functioning VHL [80].

Altogether, our results suggest that protein outlier analysis may require integration with mutational and/or mRNA expression analyses to better predict response to therapy. Additionally, mass spectrometry for protein expression can be valuable in validating RNA-seq and RPPA data as well as capturing new putative druggable events (Additional file 1, Additional file 3: Figure S2). mRNA and phosphoprotein expression outlier analysis identified potential therapeutic targets in 2559 tumors (39%).

### Integrative omics analysis of druggability

**Fig. 6:**
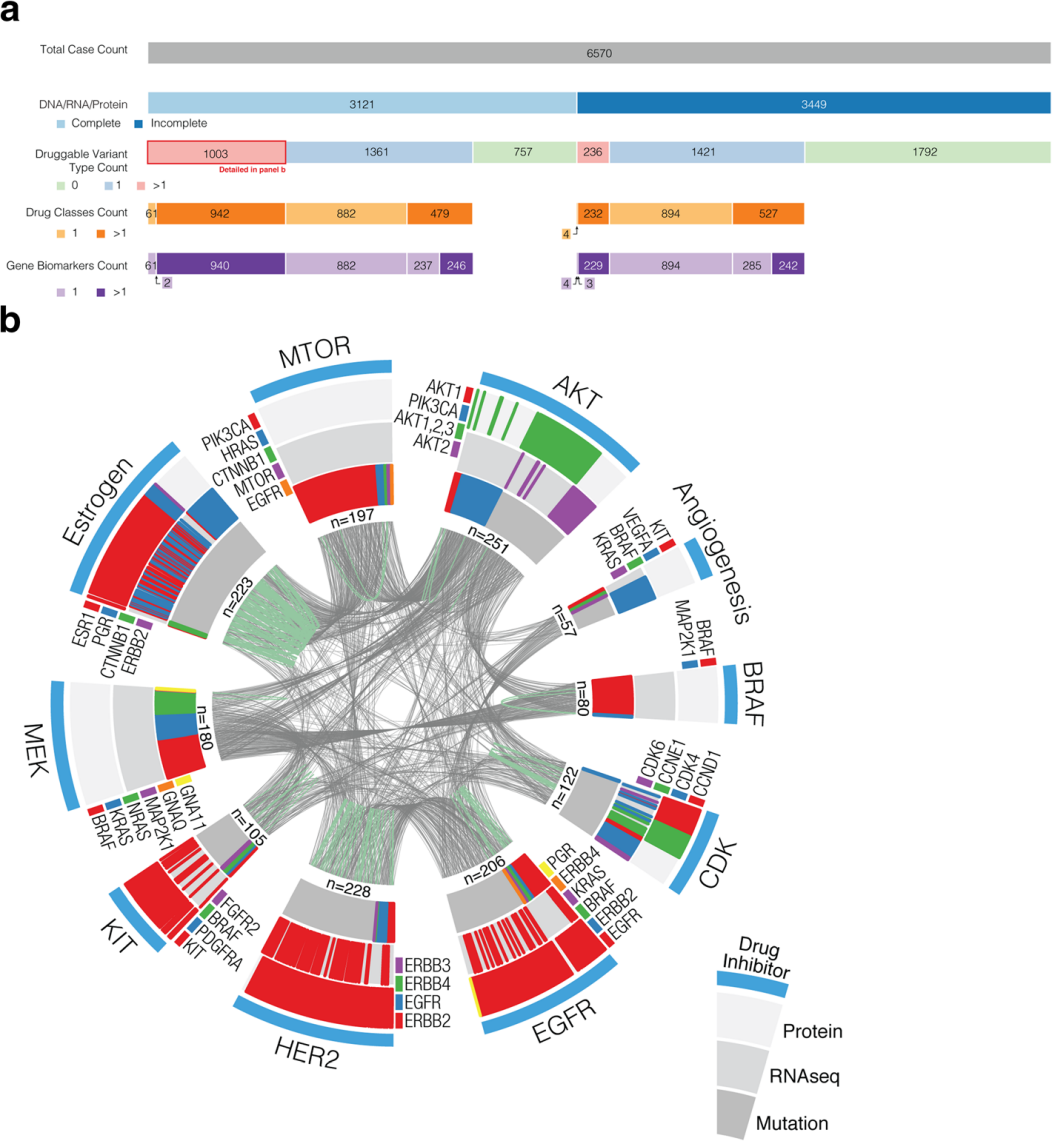
Integrative omics analysis of druggability. **a** TCGA tumor samples are sorted by completeness of DNA/RNA/protein profiling, number of variant types supporting druggability, number of drug classes, and number of druggable genes. Of the 3121 tumor samples with complete profiling, 1003 are potentially druggable based on > 1 variant types (mutational, RNA expression, protein expression) and are represented in **b**. **b** Multi-drug and multi-omic relationships within tumor samples. Ten outer sectors separate samples according to biomarkers associated with sensitivity to one of ten FDA-approved drug classes. Each outer sector consists of three tracks: DNA mutation (inner), RNA expression (middle), and protein expression (outer). Different colored bands within these tracks represent different genes whose variants implicate druggability in a single tumor sample. The genes represented in each sector vary according to drug class; adjacent to each sector is a legend indicating represented genes. The total number of unique samples is labeled under each sector. A gray link (between wedges) represents a single tumor with biomarkers associated with sensitivity to multiple drug classes. A green link (within a wedge) represents a single tumor with multiple biomarkers of the same variant type associated with sensitivity to a single drug class (e.g., a single tumor with RNA expression in *ESR1* and *PGR*)

Assessing alterations in multiple levels of data across genes may improve predictions of druggability. For example, with trastuzumab, a single testing method or biomarker (CNV, mRNA expression, protein expression, etc.) can be insufficient for stratifying patients into responders and non-responders [59]. Therefore, we assessed druggability using comprehensive mutational, RNA-seq, and RPPA data in 3121 tumors. Of these, 1003 tumors (32%) are potentially druggable based on two or more data types (genomic, transcriptomic, proteomic) (Fig. 6a, Additional file 2: Table S8), affording an opportunity for clinical or mechanistic analyses connecting drug-associated mutations with transcriptomic/proteomic expression events. Figure 6b and Additional file 2: Table S10 depict tumors with multiple levels of alterations associated with sensitivity to one of ten categories of FDA-approved cancer drugs (Additional file 2: Table S9). Seventy-two tumors had elevated mRNA and protein expression of HER2; these may be expected to have greater or more uniform sensitivity to HER2 inhibition than tumors with elevated mRNA or protein expression alone. Identifying mutations associated with drug resistance may further improve predictions of druggability. RAC1 P29S co-occurs with mutations in BRAF and MEK1 in four SKCM tumors (Additional file 2: Table S17, Additional file 3: Figure S3). RAC1 P29S renders SKCM resistant to BRAF/MEK inhibition [57]; testing for RAC1 P29S may identify patients with BRAF V600E SKCM unlikely to benefit from BRAF/MEK inhibitor. In this case, the single-gene paradigm of existing companion diagnostics may be insufficient to determine best treatment options; rather, comprehensive mutational profiling should be considered.

Multi-omics profiling also reveals opportunities for combinatorial therapy. AKT1 E17K co-occurs with BRAF V600E in five tumors (Additional file 2: Table S17, Additional file 3: Figure S3). Combining an AKT inhibitor with the current standard of treatment for BRAF V600E*-*positive SKCM (BRAF/MEK co-inhibition) may delay drug resistance [81]. Transcriptomic and proteomic expression profiling reveals 48 additional tumors with BRAF V600E/K and elevated AKT (AKT1/2/3) expression at the mRNA or protein/phosphoprotein levels; these may also benefit from BRAF/AKT inhibition (Fig. 6b, Additional file 2: Table S10). Similarly, Fig. 6b shows that 38 tumors contain biomarkers of response (i.e., mutational or expression based) for both EGFR and CDK inhibitors. Though both therapies are FDA approved, no clinical trials to date have examined combinatorial therapy with EGFR and CDK dual inhibition. Additionally, 105 tumors contain activating PIK3CA mutations co-occurring with elevated mRNA or protein expression of ESR1 or PGR. Given the success of mTOR and anti-estrogen therapy in ER-positive breast cancer [82], this combination may be useful in other cancer types that are dependent on hormonal or PI3K/mTOR signaling. By identifying tumors with biomarkers of response to multiple drugs, and by identifying variations in biomarkers across gender and ethnicity (Additional file 1, Additional file 2: Table S11, Additional file 3: Figure S4), multi-omics profiling can facilitate the rational design of clinical trials for combinatorial therapy.

### Validation of druggability analyses with large-scale drug screening

We sought to provide support for our two hypotheses that our approaches relied upon: (1) a drug with evidence supporting use in a given cancer type can be repurposed to other cancer types that contain a shared genetic alteration; (2) gene/protein expression outlier score is a predictor of drug sensitivity. To test these hypotheses, we utilized the Genomics of Drug Sensitivity in Cancer (GDSC) database, which contains drug sensitivity data for around 75,000 experiments of 138 anticancer drugs (Additional file 2: Table S4) across 700 cancer cell lines [83]. We extracted tissue type, the mutational landscape (missense mutations and in-frame indels), gene expression, and drug sensitivity information for each cell line.

**Fig. 7:**
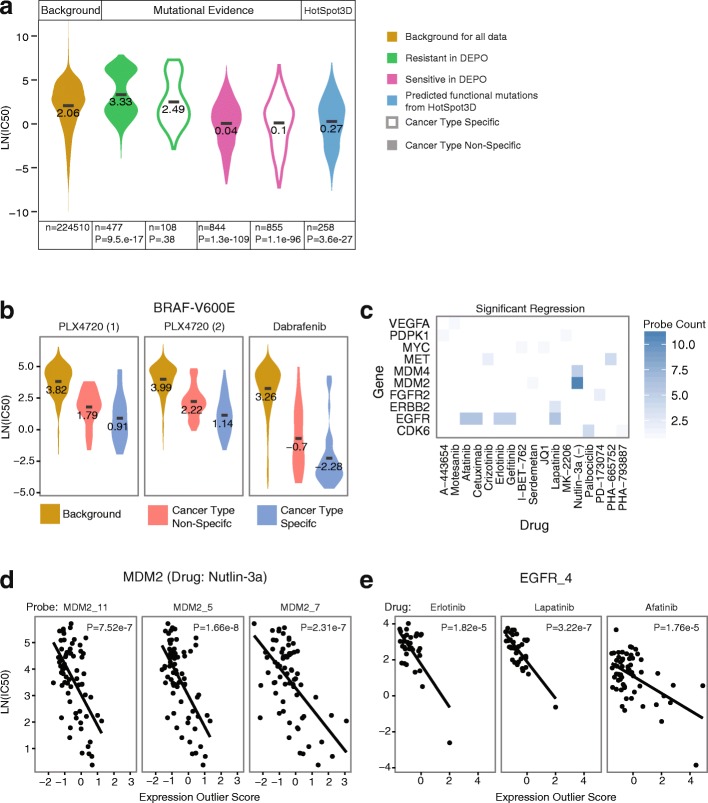
Cell line-based validation. **a** Violin plots show the distribution of drug response (*y-*axis) of cell lines with drug-associated mutations compared to the background distribution (dark yellow). The type of distribution is indicated in the top gray bar of the panel with distributions of the background, cell lines with mutations in DEPO (Mutational Evidence), and cell lines with putative functional mutations as predicted by HotSpot3D (HotSpot3D). Sensitive and resistant mutations in DEPO are indicated by a green and pink fill color, respectively. Violin plots outlined in a bold black color indicate the cancer-type-specific distribution. The bottom gray bar indicates sample size and *P* value (Mann-Whitney *U* test) for the distribution when compared to the background. **b** The distribution of drug response (*y*-axis) for three BRAF inhibitors (PLX4720 (1), PLX4720 (2), and dabrafenib) are shown. For each drug, the background distribution and drug response for cell lines with the BRAF V600E mutation in the cancer-type-specific setting and non-specific setting are shown. **c** Expression outlier scores for genes (*y*-axis) with significant negative correlation with a paired drug (*x*-axis) are shown. The intensity of shading corresponds to the number of probes that registered as significant for a gene-drug pair. **d** Scatter plots of the drug response (*y*-axis) of Nutlin-3a and expression outlier scores (*x*-axis) are shown for three different probes of MDM2. The best fit line and *P* values for the linear regression are also shown. **e** Scatter plots of the drug response (*y*-axis) to three different drugs (erlotinib, lapatinib, and afatinib) and expression outlier scores (*x*-axis) are shown for 1 probe of EGFR. The best fit line and *P* values for the linear regression are also shown

Twenty-six sensitive mutations from DEPO are found in GDSC cell lines paired with 44 drugs (Additional file 2: Table S5). BRAF V600E, PIK3CA H1047R, and KRAS G12D occur most frequently in GDSC cell lines. Overall, the mean LN(IC_50_) for cell lines that contain a sensitive mutation from DEPO was significantly lower than background LN(IC_50_) in both the cancer-type-specific and non-specific setting (Mann-Whitney *U* test, *P* = 1.1e−96 and *P* = 1.3e−109, respectively) (Fig. 7a). Individual variant/drug combinations from DEPO also performed well; 39 variant/drug combinations in the cell line data occurred in sufficient samples in both the cancer-type-specific and non-specific settings for statistical analysis (Additional file 2: Table S6). This represented 6 of 26 sensitive mutations. In both the cancer-type-specific and non-specific settings, 19 variant/drug combinations had significantly lower mean LN(IC_50_) than background LN(IC_50_) for the corresponding drug. Based on these 19 drug-variant combinations, 4 out of 6 sensitive mutations in DEPO (KRAS G12V, BRAF V600E, NRAS Q61K, and KRAS G12D) were significantly associated with sensitivity to at least one of their paired drugs in both the cancer-type-specific and non-specific settings. For example, cell lines with BRAF V600E were associated with sensitivity to BRAF inhibitors PLX4720 (1), PLX4720 (2), and dabrafenib in both the cancer-type-specific (SKCM) and non-specific settings (BRCA, COADREAD, GBM, LGG, LIHC, and THCA) (Fig. 7b). Two out of six mutations (PIK3CA H1047R and KRAS G12C) was associated with sensitivity in either the cancer-type-specific or the non-specific setting. Cell lines with PIK3CA H1047R had a significantly lower mean LN(IC_50_) in the cancer-type-non-specific setting; however, this category encompassed several cancer types, including BRCA, HNSC, and ovarian serous carcinoma (OV). Similarly, cell lines with KRAS G12C had a significant lower mean LN(IC_50_) in the cancer-type-specific setting, encompassing LIHC, LUAD, LUSC, and pancreatic adenocarcinoma (PAAD). Overall, our analyses provide some evidence to support our hypothesis that drugs can potentially be repurposed across several cancer types using shared mutational biomarkers of druggability. It must be noted, however, that sensitivity to drug response in cell lines does not necessarily translate over to clinical efficacy, and RAS- and PIK3CA-mutant cancers continue to be controversial.

To verify that gene expression outlier score was correlated with drug response, we conducted linear regression analysis for gene probe/drug combinations (Additional file 2: Table S18) using 116 different probes for 22 genes in DEPO. Forty-two probe/drug combinations corresponding to 10 genes had significant negative correlation (*P* < 0.05) between LN(IC_50_) and gene expression outlier score (Fig. 7c, Additional file 2: Table S7). For example, MDM2 expression correlates with sensitivity to nutlin-3a and EGFR expression correlates with sensitivity to erlotinib, lapatinib, and gefitinib (Fig. 7d, e). Similar trends are observed in CDK6 with palbociclib (PD-0332991: CDK4/6 inhibitor) and ERBB2 with lapatinib (Additional file 2: Table S7). Though cell line-based validation does not guarantee 100% drug response in patients, our analysis demonstrates that expression in 10 of 22 genes correlates with drug sensitivity in GDSC. Expression in other genes such as AKT2 and KIT did not correlate with drug sensitivity (Additional file 2: Table S7). However, this does not rule out the clinical utility of expression assays for these genes given that, for instance, KIT protein expression is an FDA-approved companion diagnostic for imatinib use. Overall, our analysis suggests that using gene expression outliers is a reasonable approach for predicting druggability in human cancers; however, some of these interactions still need to be validated in a clinical setting.

## Discussion

**Fig. 8:**
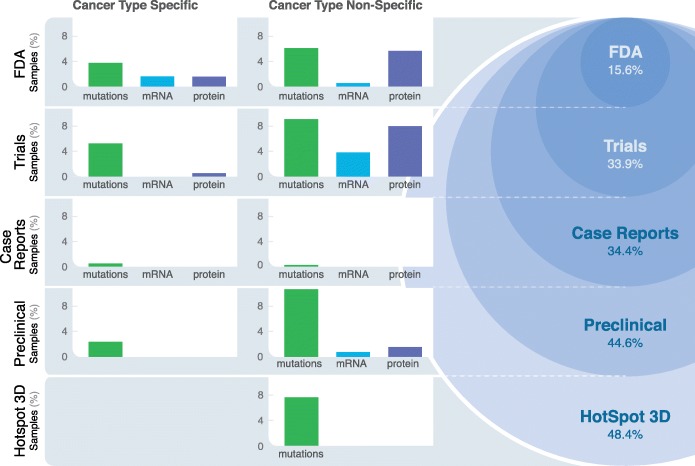
Summary of multi-omics-based druggability. Bar graphs show the percentages of tumor samples with a drug-associated variant type (mutation, mRNA expression, protein expression) in the cancer-type-specific and cancer-type-non-specific settings. The circular display shows cumulative percentages of tumor samples with drug-associated biomarkers of successively decreasing levels of evidence

This study presents a pan-cancer analysis of multi-omics-driven prescription of targeted therapy across 6570 TCGA patients. Using DEPO, a curated database of variant/drug interactions with clinically relevant annotations, we investigated the frequency of potential druggable multi-omics alterations based on various levels of evidence to help guide future clinical trials. After adjusting the percentages of potentially druggable tumors based on our validation strategy, we found that mutational, mRNA expression outliers, and phosphoprotein/protein expression outliers implicate druggability of 5% of tumors, respectively based on FDA-approved interactions only. However, up to 15.6% of the cohort could benefit if repurposing of these FDA-approved interactions to other cancer types are further explored; this percentage could increase to 33.9, 34.4, 44.6, and 48.4% of tumor samples based on clinical trials, case reports, preclinical evidence, and HotSpot3D evidence, respectively should these drug-variant interactions be approved clinically in their respective cancer types (Fig. 8, Additional file 2: Table S19, Additional file 3: Figure S5).

Our analysis illustrates the potential of a “precision oncology” approach to prescribe targeted therapy to a pan-cancer cohort of patients. Compared to prior work [17], our study offers four novel advancements. First, with DEPO, our analysis of druggability in a given tumor is exclusively based on mutation/drug interactions rather than gene/drug interactions, with variants including both predefined mutations (e.g., BRAF V600E) and categories of mutations (e.g., EGFR exon 19 deletions). The most comprehensive prior study assessing prescription of anticancer drugs included fewer than 10 mutations associated with drug sensitivity [17] (http://www.intogen.org/downloads); in comparison, the present study includes 362 mutations associated with drug sensitivity. Second, while prior studies exclusively used genomic data to infer druggability [12, 17], ours is comprehensive in its use of genomic, transcriptomic, *and* proteomic data types, specifically leveraging mRNA expression and phosphoproteomic expression data to further define tumors with potential drug-associated biomarkers. It further demonstrates that integrating data types can allow novel, personalized combinatorial therapy. Third, it uses an analytic tool to create a set of putative druggable mutations, of which a subset occurring in BRAF were tested and validated in vitro. Finally, we used a large-scale drug screening dataset (GDSC) to support our predictions of druggability based on repurposing across cancer types and expression outlier analysis. GDSC and other drug screening datasets have been used to identify biomarkers of drug sensitivity in hypothesis-free analyses [18, 84, 85], but our study is unique in using GDSC as orthogonal validation of putative biomarkers from clinical trials, case reports, and preclinical studies.

Though our study and prior studies [12, 15, 17] implicate large percentages of tumors as potentially druggable (48% and 94%/76%/73%, respectively), prior studies made several assumptions regarding off-variant and off-target drug activity that may not be clinically feasible. For example, using the more stringent prescription guidelines of the present study (variant/drug prescription with no off-variant or off-target effects), only 12.3% of tumors in Rubio-Perez et al. would be druggable. Furthermore, ongoing clinical trials [86, 87] argue that more accurate druggability annotations require specifying alterations at the variant level, as the present study does, but which Frampton et al. [15] and Van Allen et al. [12] do not. Realistically, only a fraction of the 48% of tumors with potential drug-associated omics alterations will be clinically druggable because the mere presence of a shared genetic biomarker (mutation, mRNA/protein expression outlier) does not guarantee clinical efficacy across cancer types, nor does it guarantee acceptable clinical toxicity. Not all preclinical drug-biomarker pairs, including those predicted with HotSpot3D, will advance to clinical trials. Further, we recognize that our computational survey of the landscape of potential drug-associated omics alterations may include some controversial drug/biomarker relationships (e.g., PI3K inhibitors in PIK3CA-mutant cancers), some of which have either failed clinical trials and/or are still being actively developed in clinical trials. Nonetheless, our study is important in identifying which drug-biomarker pairs, repurposing events, and combinatorial therapies are worth exploring and provides a robust platform for both design and analysis of clinical trials.

Our analysis has several limitations. First, TCGA tumor samples are treatment naïve. Given that targeted therapy is often used once other therapeutic options (e.g., cytotoxic chemotherapy, radiotherapy) have been exhausted, tumors treated in the clinical setting may have different genomic profiles than those in this study. Second, our analysis does not account for clonal heterogeneity, which is not unreasonable given that therapies targeting genomic alterations with high variant allele frequencies can induce substantial tumor regression [88]. However, we acknowledge that for clonally heterogeneous cancer types such as GBM, even if the dominant clone is sensitive to therapy, one or more subclones lacking a druggable genomic event may escape [89]. Third, some potential expression outliers may be missed since we do not compute cancer-specific expression outliers; therefore, outliers in cancer types with low overall expression may not be identified, and only high confidence outliers that are most likely targetable are reported. Additionally, some outliers may represent cancer lineage markers or non-cancer cells within tumors and not necessarily a somatically altered pathway, such as the 58% of KICH expressing KIT (Fig. 5a). Future studies can determine which kinase expression outliers are contributing to a somatically altered pathway by checking phosphorylation and/or expression of downstream substrates. Fourth, our analysis does not consider germline mutations that sensitize a tumor to targeted therapy, nor does it attempt to use integrative omics data to predict sensitivity to immune checkpoint inhibitors. Finally, our analysis ignores therapeutic toxicity. In particular, toxicity is often a limiting factor for combination therapy [90, 91], though rationally designed combinations can reduce toxicity [92].

## Conclusions

This study is the first to comprehensively profile the druggability of cancer types using integrative omics TCGA data. While multi-omics-driven prescription of anticancer drugs is a powerful concept [17], the efficacy of each drug still requires testing within the context of clinical trials. By describing the landscape of potentially druggable alterations across cancer types, our study serves as a roadmap for the interpretation and design of clinical trials in precision oncology.

## Additional files

Additional file 1: Supplementary Materials and Methods. (PDF 33 kb)

Additional file 2: **Table S1.** Drug classes in DEPO (database of evidence for precision oncology). **Table S2.** Sensitive druggable mutations in 6570 TCGA tumors. **Table S3.** Highest level of evidence present per variant for both resistant and sensitive. **Table S4.** Drugs represented in the GDSC Cell Line Data. **Table S5.** Cell lines that contain a sensitive mutation in DEPO. **Table S6.** Mann-Whitney *U* test of distribution of Ln(IC50) values in cell lines with DEPO sensitive mutations against background distribution for each drug. **Table S7.** Linear regression statistics for probe-drug pairs. **Table S8.** TCGA tumors (out of 3121) that are druggable based on two or more variant types (genomic, transcriptomic, proteomic). **Table S9.** Ten FDA-approved drug classes. **Table S10.** TCGA tumors that are druggable with one of ten classes of FDA-approved cancer drugs based on two or more variant types (genomic, transcriptomic, proteomic). **Table S11.** Druggability and demographics. **Table S12.** Cancer types responsible for the levels of evidence in the cancer type non-specific setting for Fig. 2a. **Table S13.** Novel druggable mutations clustering with known druggable mutations identified using HotSpot3D, a proximity-based clustering tool. **Table S14.** RNA-seq data and protein RPPA data for 6366 and 3877 TCGA tumors, respectively. **Table S15.** Druggable fusions in TCGA samples. **Table S16.** Evidence to support repurposing of proteogenomic alterations across cancer types. **Table S17.** Co-occurring druggable mutations. **Table S18.** Gene expression outlier scores and drug response for all cell lines. **Table S19.** TCGA tumors (out of 6570) that are druggable based on atleast one variant (genomic, transcriptomic, proteomic). (.xlsx 2.1 MB) (XLSX 2039 kb)

Additional file 3: **Figure S1.** Fusions in the TCGA cohort. **Figure S2.** Druggable protein expression outliers using mass spectrometry. **Figure S3.** Co-occurring druggable mutations represent opportunities for combinational and alternative therapy. **Figure S4.** Druggability and Demographics. **Figure S5.** Potential Druggability by Cancer Type. (PDF 514 KB) (PDF 501 kb)

## Abbreviations

ACC: Adrenocortical carcinoma
AML/LAML: Acute myeloid leukemia
BLCA: Bladder urothelial carcinoma
BRCA: Breast adenocarcinoma
CESC: Cervical squamous cell carcinoma and endocervical adenocarcinoma
CNA: Copy number amplification
CNL: Copy number loss
CNV: Copy number variation
COADREAD: Colon and rectal carcinoma
CPTAC: Clinical Proteomic Tumor Analysis Consortium
DCC: Data Coordinating Center
DEPO: Database of Evidence for Precision Oncology
FBS: Fetal bovine serum
FDA: Food and Drug Administration
FFPE: Formalin-fixed, paraffin-embedded
GBM: Glioblastoma multiforme
GDAC: Genome Data Analysis Centers
GDSC: Genomics of Drug Sensitivity in Cancer
HNSC: Head and neck squamous cell carcinoma
IQR: Interquartile range
KICH: Kidney chromophobe
KIRC: Kidney renal clear cell carcinoma
KIRP: Kidney renal papillary cell carcinoma
LGG: Low-grade glioma
LIHC: Liver hepatocellular carcinoma
LUAD: Lung adenocarcinoma
LUSC: Lung squamous cell carcinoma
MAF: Mutation annotation file
NGS: Next-generation sequencing
NSCLC: Non-small cell lung cancer
OV: Ovarian serous carcinoma
PNNL: Pacific Northwest National Laboratory
PRAD: Prostate adenocarcinoma
RBN: Replicates-based normalization
RPPA: Reverse phase protein array
SKCM: Skin cutaneous melanoma
SNP: Single nucleotide polymorphism
STAD: Stomach adenocarcinoma
STR: Short tandem repeat
TCGA: The Cancer Genome Atlas
TCPA: The Cancer Protein Atlas
THCA: Thyroid carcinoma
TKI: Tyrosine kinase inhibitor
UCEC: Uterine corpus endometrial carcinoma
UCS: Uterine carcinosarcoma
VCF: Variant call format
