# Supplementary material for: Integrative omics analyses broaden treatment targets in human cancer
Source: Genome Med. 2018 Jul 27;10:60. doi: 10.1186/s13073-018-0564-z (PMC6064051; doi:10.1186/s13073-018-0564-z)
Supplement: Supplementary file 1 — Supplementary Materials and Methods. (PDF 33 kb) [file 13073_2018_564_MOESM1_ESM.pdf]

Supplementary Materials and Methods

Technical Aspects of Clinical Proteogenomics

Clinical implementation of routine whole-exome sequencing (WES) and other proteogenomic technologies involve several technical considerations. First, quality of nucleic acid & protein extraction depend on age of tissue, fixation time, and size of specimen [96]. For example, TCGA sequencing efforts use fresh frozen tissue. Archival or FFPE tissue samples are more common in the clinical setting [97] and the quality of next-generation sequencing data generated from FFPE tissue can be less consistent. Second, tumor purity (due to stroma, necrosis, etc.), particularly in ovarian cancer, can affect fidelity. Laser capture microdissection can increase tumor purity, but it is labor-intensive and may degrade nucleic acids. Third, datasets from routine NGS of advanced cancers can be very large [98] and may require compression or cloud storage, though this may be less of an issue for NGS of targeted gene panels than for tumor exomes. Finally, nucleic acid & protein extraction, purification, characterization, bioinformatics analysis, and interpretation must be streamlined with a rapid turnaround time, particularly for patients with advanced cancers being considered for therapeutic agents.

Druggability with a “Mutation/Drug” vs “Gene/Drug” Approach

DGIdb is a tool that links drugs to genes by drawing information from multiple databases. However, druggability varies depending on the specific mutation present in a gene. Further, genes can be altered in many other ways that affect druggability (methylation, copy-number, RNA expression, protein expression). Specific gene alterations linked to druggability are not captured in DGIdb,[22, 99] meaning it is different in kind from DEPO. For example, although 393 genes in DGIdb are linked to an anti-neoplastic drug,[22] far more than the 168 genes in DEPO, DGIdb does not provide necessary information to conduct the level of analysis in the present study. We illustrate this difference using *EGFR*. A gene/drug approach, in which all *EGFR* missense and simple indels mutations are considered druggable, implicates 13%, 3%, and 26% of LUAD, LUSC, and GBM as druggable, respectively. The present study uses a mutation/drug approach to implicate druggability with EGFR inhibitors in 10%, 1%, and 11% of LUAD, LUSC, and GBM, respectively. A gene/drug approach systematically overestimates druggability, and therefore, DGIdb is insufficient for the purposes of the present study.

Proteomic Analysis with CPTAC

Additionally, to validate the findings suggested by RNA-seq and RPPA data in TCGA, we utilized mass spectrometry data from the Clinical Proteome Tumor Analysis Consortium’s (CPTAC) characterization of 3 cancer types: BRCA, OV, and COADREAD. Of 251 CPTAC tumors, 83 (33%) had elevated proteomic expression or phosphoproteomic activity suggesting druggability. This outlier detection strategy suggested potential druggability in 47%, 27% and 27% of BRCA, OV, and COADREAD tumors, respectively (**Additional File 4: Fig. S2**).

176 of 251 CPTAC tumors overlapped with the 6,570 TCGA tumors in this study. Mass spectrometric protein expression validated elevated mRNA and/or protein expression from RNA-seq and RPPA data in 21 tumors, including elevated ERBB2 expression in 11 tumors. Notably, for 45 tumors characterized by TCGA and CPTAC, mass spectrometry identified elevated expression of a druggable gene that was not corroborated by RNA-seq and RPPA. Discordance between mass spectrometry, RNA-seq, and RPPA may be due to biological variability in RNA and protein expression, or technical limitations in proteomic profiling technologies, or both.

Druggability and Demographics

We conducted an analysis of druggable biomarker prevalence as a function of sex and ethnicity (**Additional File 4: Fig. S4**). When we compared the prevalence of druggable biomarkers between male and female, we found that elevated ERBB2 and EGFR phosphoprotein expression is more common in men with BLCA than women with BLCA. PIK3CA mutations tended to be more common in women than men, regardless of cancer type. However, none of these differences are statistically significant.

For ethnicity-based analysis, we find significant differences in biomarker prevalence between Caucasians and Asians and Caucasians and African-Americans (**Additional File 2: Table S11**). Elevated CCND1 and CCNE1 phosphoprotein expression is less common in Asians and African-Americans with STAD than Caucasians, respectively. In BRCA, elevated PGR RNA expression is less common in Asians and African-Americans than Caucasians, whereas elevated ERBB2 phosphoprotein expression is more prevalent in Asians. These differences suggest that, without routine biomarker profiling, minorities may respond differently than Caucasians to both experimental therapies (e.g. CDK inhibitors in STAD) and first-line therapies (e.g. hormonal therapy in BRCA).
