# Supplementary material for: Integrative omics analyses broaden treatment targets in human cancer
Source: Genome Med. 2018 Jul 27;10:60. doi: 10.1186/s13073-018-0564-z (PMC6064051; doi:10.1186/s13073-018-0564-z)
Supplement: Supplementary file 3 — Figure S1. Fusions in the TCGA cohort. Figure S2. Druggable protein expression outliers using mass spectrometry. Figure S3. Co-occurring druggable mutations represent opportunities for combinational and alternative therapy. Figure S4. Druggability and Demographics. Figure S5. Potential Druggability by Cancer Type. (PDF 514 KB) (PDF 501 kb) [file 13073_2018_564_MOESM3_ESM.pdf]

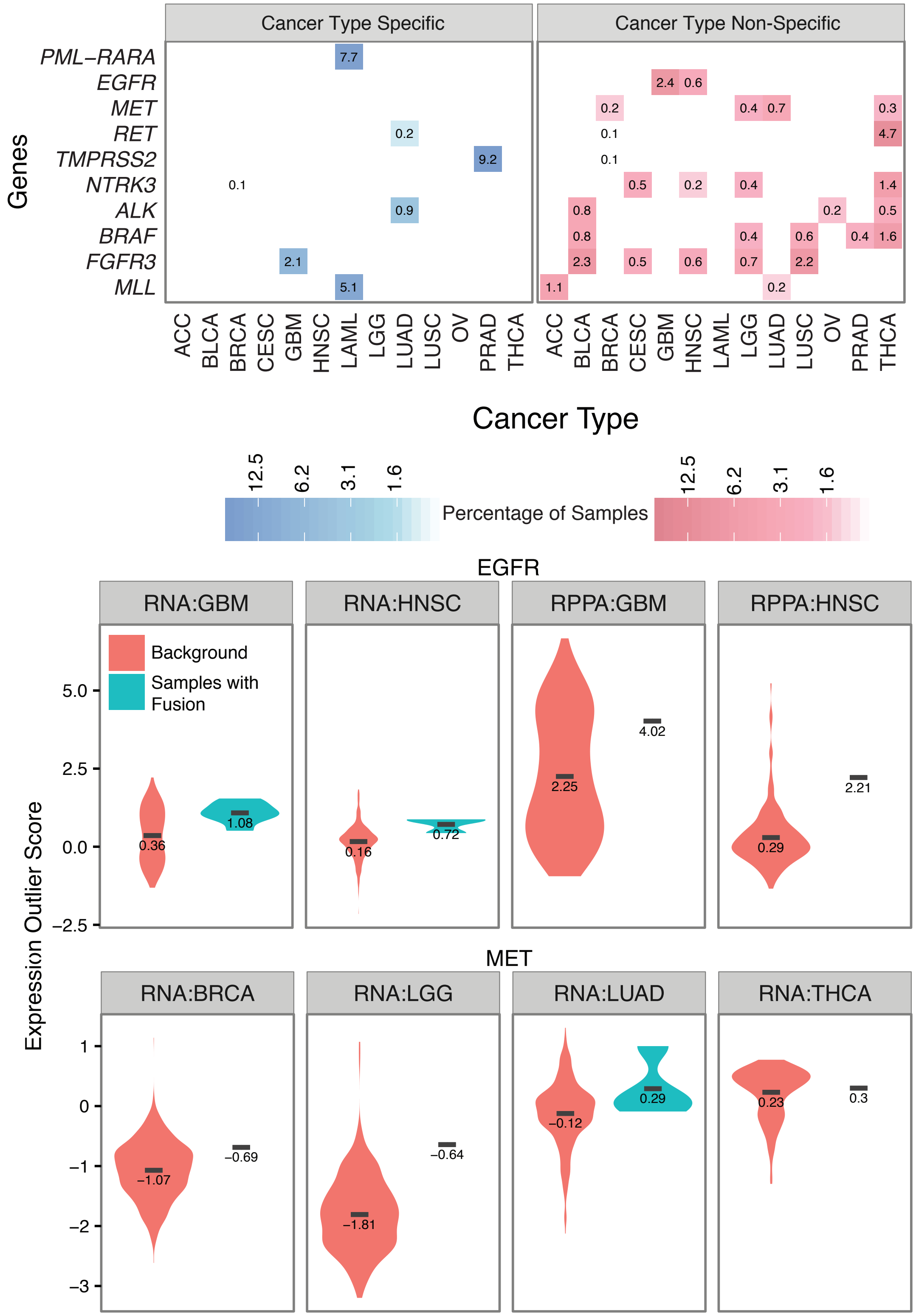


**Fig. S1. Fusions in the TCGA cohort.** a) Fraction of tumors (intensity of shading) for a given cancer type (x-axis) containing a druggable fusion from a specific gene (y-axis) in both the cancer type specific (blue) and cancer type non-specific settings (red). b) Violin plots show the distribution of expression outlier scores for samples containing fusions compared to the background distribution for both EGFR and MET. The dataset, whether RNA-seq or RPPA data, and cancer types are indicated in the gray bars above the violin plots.

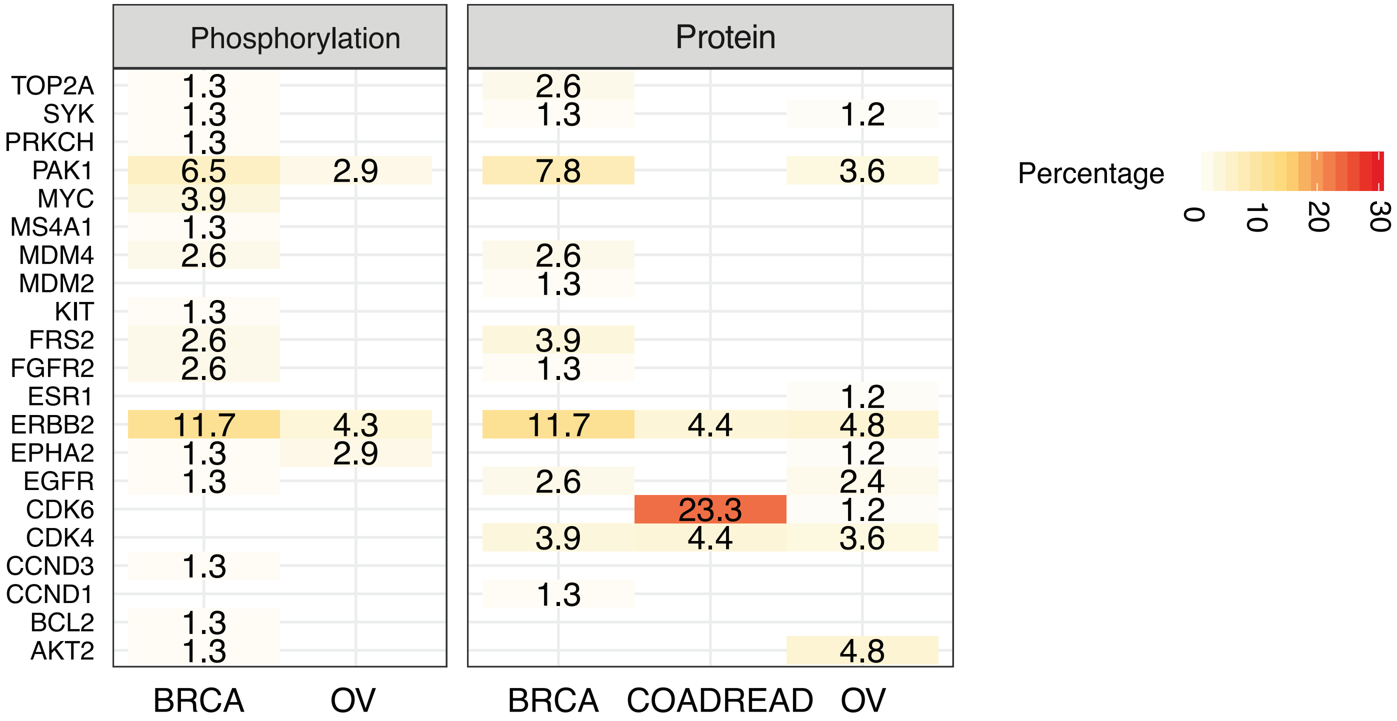


**Fig. S2. Druggable protein expression outliers using mass spectrometry.** Outlier expression analysis for proteins and its phosphorylation sites. Intensity of shading corresponds to percentage of tumor samples in a specific cancer type (x-axis) that has outlier expression in a specific gene (y-axis). The scale is limited to 30%; any percentage higher than this will be displayed as the same color. ‘Phosphorylation’ refers to expression outliers at phosphorylation sites and ‘Protein’ refers to protein expression outliers.

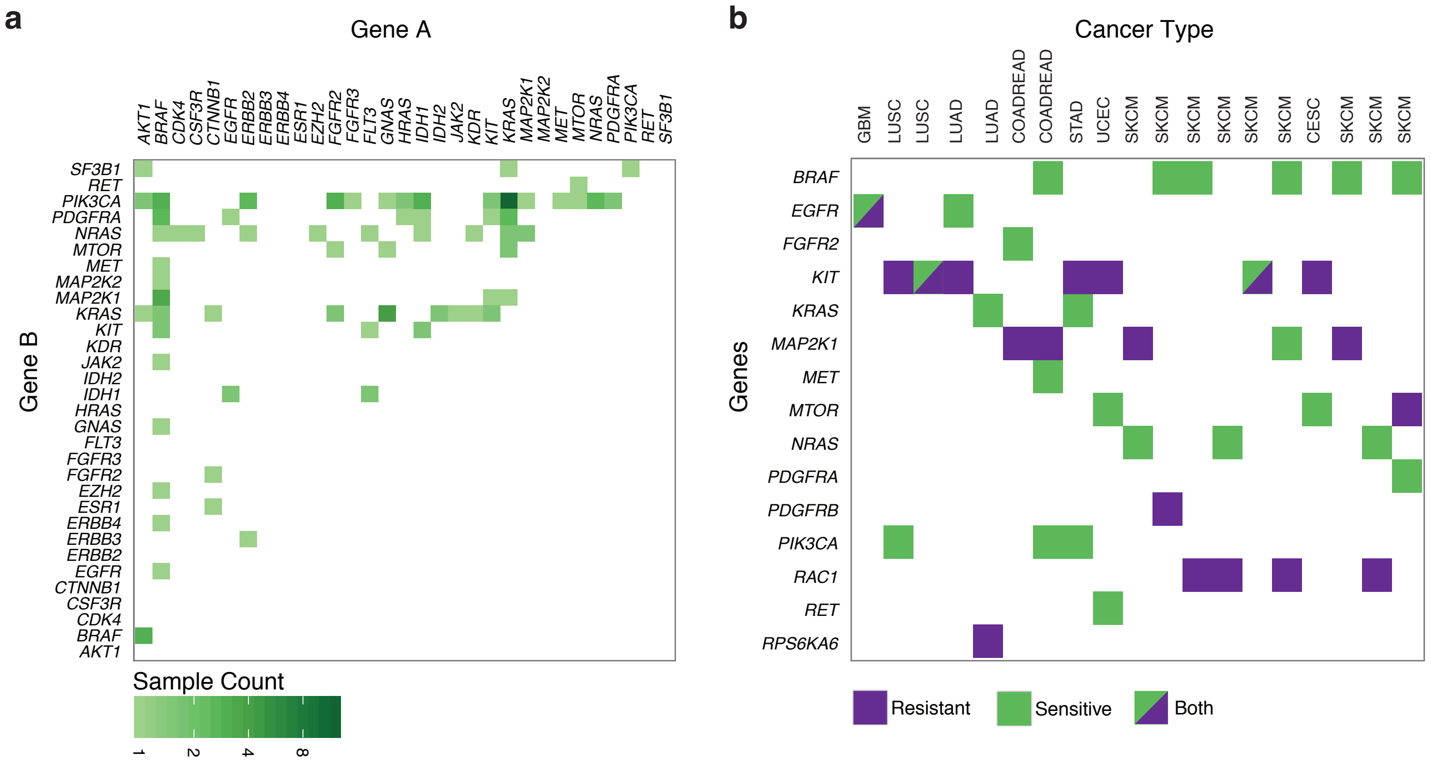


**Fig. S3. Co-occurring druggable mutations represent opportunities for combinational and alternative therapy.** a) Co-occurring mutations in TCGA tumor samples associated with drug sensitivity, with intensity of shading corresponding to the number of tumors in which a combination of co-occurring mutations occurs. Each combination is broken down into all possible gene pairs for visualization. b) Co-occurring mutations in TCGA tumors associated with drug sensitivity (green), resistance (purple), or both. Genes are represented on the y-axis. Each column represents a distinct TCGA tumor containing co-occurring mutations with cancer type labeled on the x-axis.

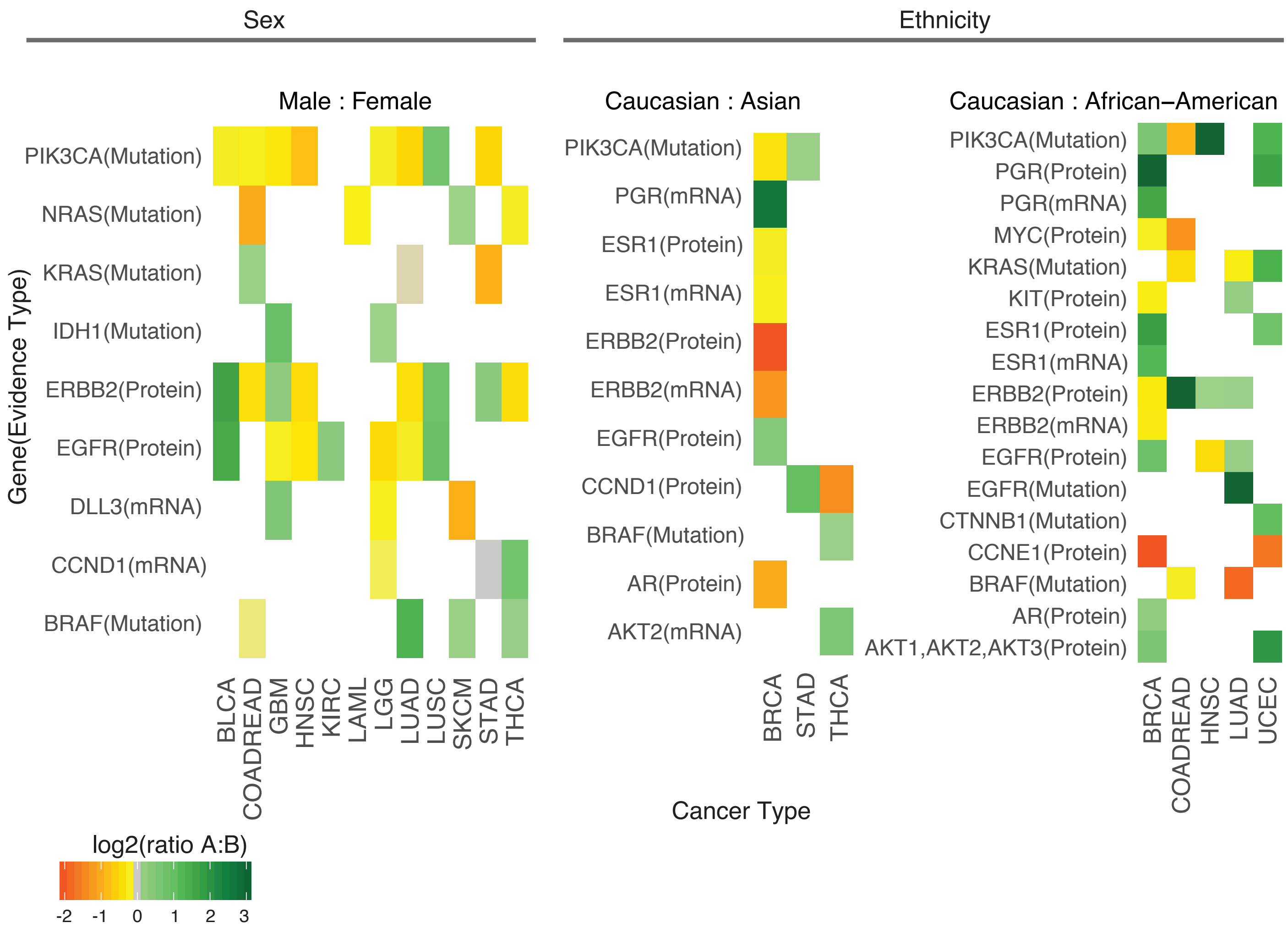


**Fig. S4. Druggability and demographics.** Sex and ethnicity variations in prevalence of biomarkers for druggability at the mutational, mRNA and protein overexpression levels (y-axis) are displayed across cancer types (x-axis). The colors in each heatmap correspond to the log2 of the prevalence of a druggable biomarker in population A divided by the prevalence of a druggable biomarker in population B. Male to female prevalence, Caucasian to Asian prevalence, and Caucasian to African-American prevalence is compared in the leftmost, middle, and rightmost heat maps, respectively.

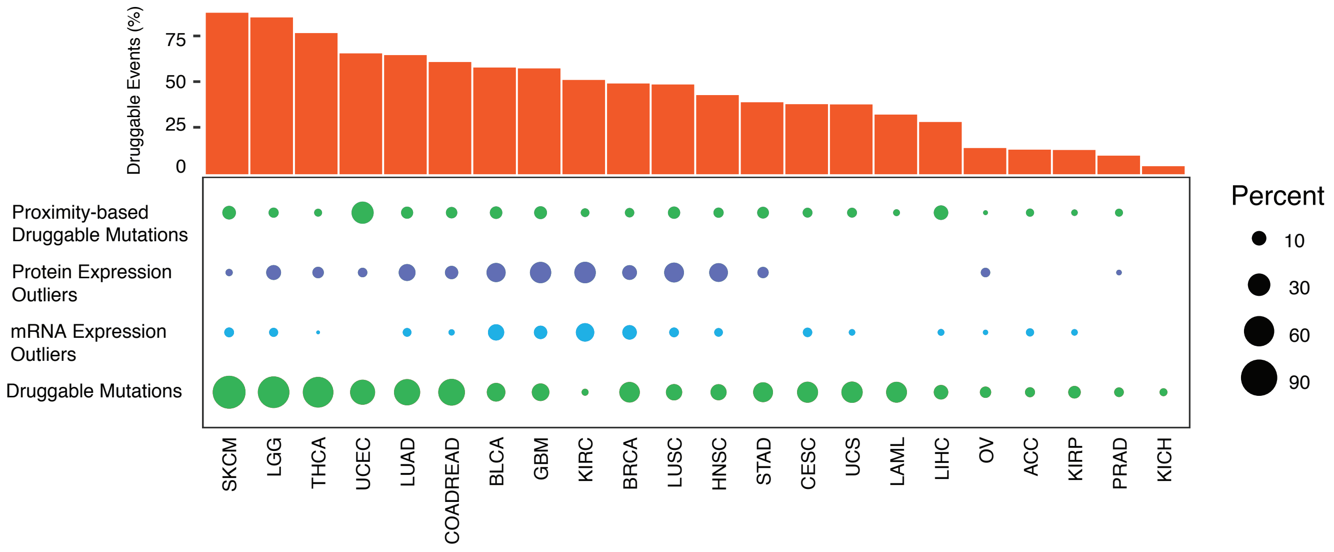


**Fig S5. Potential Druggability by Cancer Type.** The size of the bubbles indicates the fraction of samples in each cancer type (x-axis) that may be druggable based on each of the four genomic and proteomic variant types implicating druggability (y-axis). The bar graph indicates the total percentage of potentially druggable samples by cancer type based on all four genomic and proteomic variant types.
